# Mapping Randomized Controlled Trials of Family- and Community-Centered Healthcare Interventions for Pediatric Asthma, Type 1 Diabetes, or Juvenile Idiopathic Arthritis—A Scoping Review

**DOI:** 10.3390/children13091232

**Published:** 2026-09-11

**Authors:** Svetlana Solgaard Nielsen, Alessio Bricca, Stavros Orologas, Ann-Marie Malby Schoos, Anna-Helene Bohr, Kija Lin Østergaard, Julie Mondahl, Sofie Ahlgreen Gram, Dan Grabowski, Fatma Demircioglu Bilgin, Marie Sofie Oxlund Hoppe, Tine Petz Rasmussen, Charlotte Simonÿ, Alexander Luijk, Line Nørgaard Remmen, Henrik Hallas, Louisa Mittet, Mette Tækker Jensen, Jeanette Reffstrup Christensen

**Affiliations:** 1 The Research and Implementation Unit PROgrez, Department of Physiotherapy and Occupational Therapy, Central and West Zealand Hospital, 4200 Slagelse, Denmark; klo@regionsjaelland.dk (K.L.Ø.); jumo@regionsjaelland.dk (J.M.); cpsi@regionsjaelland.dk (C.S.); 2 Department of Regional Health Research, University of Southern Denmark, 5230 Odense, Denmark; 3 Center for Muscle and Joint Health, Department of Sports Science and Clinical Biomechanics, University of Southern Denmark, 5230 Odense, Denmark; abricca@health.sdu.dk; 4 Department of Health Sciences, European University Cyprus, Nicosia 1516, Cyprus; s.orologas@euc.ac.cy; 5 Department of Pediatrics, Central and Western Zealand Hospital, 4200 Slagelse, Denmark; ann-marie.schoos@dbac.dk (A.-M.M.S.); agbo@regionsjaelland.dk (A.-H.B.); henrik.leth.hallas@regionh.dk (H.H.); 6 Department of Pediatrics, Copenhagen University Hospital, Amager and Hvidovre Hospital, 2650 Hvidovre, Denmark; 7 Copenhagen Prospective Studies on Asthma in Childhood (COPSAC), Copenhagen University Hospital, Herlev and Gentofte Hospital, 2200 Copenhagen, Denmark; 8 Department of Clinical Medicine, University of Copenhagen, 2200 Copenhagen, Denmark; 9 Department of Gynecology and Obstetrics, Zealand University Hospital, 4000 Roskilde, Denmark; 10 The Research Department, Central and West Zealand Hospital, 4200 Slagelse, Denmark; 11 Sangens Hus (The House of Song), 7400 Herning, Denmark; 12 Copenhagen University Hospital, Steno Diabetes Center Copenhagen, Department of Prevention, Health Promotion and Community Care, 2730 Herlev, Denmark; dan.grabowski@regionh.dk; 13 Department of Public Health, University of Southern Denmark, 5230 Odense, Denmark; 14 Department of Gynecology and Obstetrics, Odense University Hospital (OUH), 5000 Odense, Denmark; 15 Research Unit of General Practice, 8000 Aarhus, Denmarktipras@rm.dk (T.P.R.); lnre@ucsyd.dk (L.N.R.); lmitt@regionsjaelland.dk (L.M.); jrchristensen@health.sdu.dk (J.R.C.); 16 Centre for Health Research, Zealand University Hospital, Nykøbing F, 4000 Roskilde, Denmark; alelu@regionsjaelland.dk; 17 Occupational Therapy Programme, University College South Denmark, 6705 Esbjerg, Denmark; 18 Driven, Research Center for Motivation and Behavior Science, Department of Sport and Biomechanics, Faculty of Health Sciences, University of Southern Denmark, 5230 Odense, Denmark; 19 Research Unit of General Practice, Department of Public Health, Faculty of Health Sciences, University of Southern Denmark, 5230 Odense, Denmark

**Keywords:** adolescents, children, community, family health promotion

## Abstract

**Highlights:**

**What are the main findings?**

- Family- and community-centered care is mainly delivered through short-term, multicomponent interventions, with a strong emphasis on populations with asthma.
- While the interventions included typically resulted in disease-related and clinical outcomes, outcomes related to participation in everyday life, school functioning, and family well-being were less frequently considered.

**What are the implications of the main findings?**

- The findings highlight the need for broader, diagnosis-inclusive approaches and stronger involvement of community stakeholders and policymakers in future family- and community-centered interventions.
- Evaluation of the interventions’ societal impacts and mechanisms of change will improve the evidence base of family- and community-centered care.

**Abstract:**

Background: Family and community are key support sources for children and adolescents with chronic illnesses. This study aimed to map the characteristics of family- and community-centered interventions for pediatric chronic somatic disease. Methods: This scoping review followed the Arksey and O’Malley guidance. Through a systematic search in Medline (PubMed), EMBASE, PsycINFO, Cochrane, and CINAHL, free web sources, the gray literature, and citations, we identified publications of randomized controlled trials evaluating family- and community-centered interventions for children aged 0–17 years with asthma, type 1 diabetes, or juvenile idiopathic arthritis. Two or more reviewers independently conducted screening, verification of machine-assisted data extraction, risk-of-bias assessment, and narrative synthesis. Results: Of 7285 items identified, we included 76 publications on 71 interventions comprising 16,342 participants (median n = 157; IQR 81–303; range 12–1316) with a median age of 9 (range 0–19) years. Interventions incorporating educational, psychosocial, and care-coordination elements targeted asthma (68%), type 1 diabetes (29%), and juvenile idiopathic arthritis (3%) that had lasted ≤6 months (63%) and were delivered in person (83%) by healthcare professionals (62%). Primary outcomes included symptom burden (44%), coping with the disease (24%), disease control (20%), activity participation, child psychosocial functioning, medication or healthcare use (18% each), or child quality of life (17%). Usual care (59%) and other treatment (31%) were frequent comparators. Conclusions: Most interventions were short-term, healthcare professional-led multicomponent programs compared with usual care. Primary outcomes targeted symptom management and disease control. Mixed diagnoses, peer support, family activities, and non-professional delivery were uncommon. This study identified gaps in diagnosis coverage and the need for additional high-quality studies.

## 1. Introduction

Asthma, type 1 diabetes (T1D), and juvenile idiopathic arthritis (JIA) are among the most common chronic diseases affecting children and adolescents [1,2,3,4]. In Denmark, approximately 95,000 (11.7%) children and adolescents live with asthma [3,5], 3500 (0.3%) with T1D [6], and 1200 (0.1%) with JIA [7]. All three health conditions are lifelong or long-term diseases that require ongoing management and can place a substantial burden on children, adolescents, and their families [8,9,10].

Although current treatments of asthma, T1D, and JIA are solidly established, there is still need for approaches that bring their well-being up to a similar level with their peers [11,12,13]. Recent reports of family and community involvement in such interventions are promising. For example, the World Health Organization (WHO) has highlighted the importance of delivering preventive interventions close to where people live, learn, and play to address obesity, asthma, and other chronic health conditions in children and adolescents [14]. A narrative summary of ten reviews of interventions targeting different aspects of health in school-aged children highlighted the school as an appropriate platform for intervention delivery and implementation of health promotion policies [15]. At the same time, results from another systematic review with a meta-analysis of 25 psychoeducative intervention studies for children with chronic conditions was inconclusive regarding the mode of the delivery (e.g., in-clinic, at home, or in school) [16].

A recent rapid scoping review by Chow et al. [17] identified 63 relevant publications with various study designs on family-centered interventions for children with chronic diseases that were published in 2019–2020. Of those, six studies included children with asthma, T1D, or JIA, including two that documented community involvement. The authors described the types of activities and processes of the interventions and highlighted the need for more rigorous stakeholder involvement in intervention development and greater attention to siblings’ needs. However, the conclusions were based on studies published within a limited timeframe, referring to both somatic and mental health conditions, and excluding adolescents aged 13–18 years. Another systematic review and meta-analysis including six published and two unpublished sources spanned children and adolescents aged 0–21 years with diverse chronic conditions, including asthma and T1D, concluded that family-focused, community-based approach could support family functioning [18]. Particularly, intervention components, such as guided joint role-playing exercises, psychoeducation, and inter-relational development, were useful. However, the conclusions of this study were based on populations with limited representation of, i.e., asthma, or interventions targeting parents. Therefore, there is a need to systematically map and characterize randomized controlled trials of family- and community-centered interventions targeting children with asthma, T1D, and JIA. This review can identify existing evidence, highlight gaps in current intervention research, and inform the development of future family-centered approaches for pediatric chronic disease management.

The objective of this study is to provide an overview of intervention, comparator and outcome characteristics of trials investigating community- and family-centered healthcare interventions for children and adolescents living with asthma, T1D, or JIA.

## 2. Methods

### 2.1. Data Sources and Search Strategy

This scoping review, registered in the Open Science Framework (OSF, ID B9njr), was conducted in accordance with the methodology proposed by Arksey & O’Malley, providing a systematic and rigorous evidence mapping approach [19]. Community-centered interventions were defined as interventions engaging local actors, settings, or systems beyond the clinical environment, as described by South et al. [20]. These interventions may pursue strengthening communities (e.g., by a greater awareness and infrastructure), building-up volunteer and peer support, collaborations and partnerships (e.g., in co-production and planning), and widening access to community resources (e.g., through increased service capacity, new referral pathways) [20]. As telehealth can improve access, reduce costs, and support local community health, telehealth solutions applicable for use outside clinical settings, e.g., at home or school, were considered community-based [21]. Family-centered interventions are defined as those involving family members in care planning, decision-making, and support, according to Kokorelias et al. [22]. These interventions may focus on collaboration between family members and healthcare providers, consideration of family contexts, educating patients, families, and health professionals, and policymaking [22].

The search strategy aimed to identify both published and unpublished studies. An initial limited search of MEDLINE (through PubMed) was undertaken to identify articles on the topic. Text words from the titles, abstracts, and keywords of relevant articles, as well as the index terms, were used to develop a full block search strategy in MEDLINE (PubMed), EMBASE, PsycINFO, Cochrane, and CINAHL. The search strategy included separate diagnosis-specific search blocks for asthma, T1D and JIA. Within each database, these blocks were combined with the family and community concepts using database-specific controlled vocabulary and free-text terms. The block search strategy applied Boolean operators ‘AND’ and ‘OR’ to combine the topics of interest: children/adolescents AND family AND community AND diagnosis AND outcomes of interest. Each block included all identified keywords and index terms adapted to each database (see literature search strategy in Supplementary Materials). The last literature search was performed on 17 September 2025. Relevant systematic reviews detected during the literature search were inspected for individual studies eligible for this scoping review. The first three pages of free search on Google Scholar were inspected for relevant sources. A search in the Open Grey database helped identify unpublished studies and the gray literature. The reference lists of all included publications were screened for additional studies.

### 2.2. Review Questions

We followed the PCC (Participants, Concept, Context) framework to formulate the main research question [23]: Which interventions, comparators and outcome characteristics are used in family-centered community healthcare interventions for children and adolescents living with asthma, T1D, or JIA, and their families?

### 2.3. Inclusion and Exclusion Criteria

Full-text study reports were included if they met the following inclusion and exclusion criteria (Table 1).

Studies including participants older than 17 years were considered eligible when data for participants within the study population were predominantly within the eligible age range. We considered potential bias resulting from the inclusion of these interventions less problematic than the potential loss of relevant information that would have resulted from their exclusion.

### 2.4. Study Selection

Following the search, all identified citations were collated and uploaded into EndNote version 21.5 (Clarivate Analytics, Philadelphia, PA, USA; University of Southern Denmark license) and duplicates were removed. Deduplicated citations were transferred to Covidence software (University of Southern Denmark license), which automatically repeated deduplication, finding additional duplicates. At least two independent reviewers screened the titles and abstracts of all retrieved records to assess their eligibility according to the review’s inclusion and exclusion criteria and remove any additional duplicates.

Potentially relevant items were retrieved in full text and assessed in detail against the inclusion and exclusion criteria by two or more independent reviewers collaborating in pairs. Two authors of brief reports were contacted with a request for a full-text publication of the results but did not respond. Reasons for exclusion were noted in Covidence and reported in the review’s flow chart. Any disagreements that arose at each stage of the selection process were resolved through discussion and involving a third reviewer (JRC), if needed.

### 2.5. Data Extraction

A data extraction form in Microsoft Excel was developed specifically for this study by SSN to extract all relevant information from the sample studies to answer the research question [20,22]. SSN piloted the data extraction form with the first five studies from the sample, and then modified and revised the form by adding the columns: Intervention Intensity and Dose, Harms and Adverse Events, and Community Anchoring. The first round of data extraction was performed by Microsoft 365 Copilot, GPT-5 chat model, and supervised by SSN, who also performed the first verification of the output. Then, another independent researcher from the author group performed a second verification round of the output. We recorded all modifications made during human review. Human verification resulted in minor amendments to the initial machine-assisted extraction for n = 43/76 (57%) of publications, primarily to harmonize descriptive variables across studies and calculate information not explicitly reported by authors, including participant age, intervention duration, sample size, community anchoring, and outcome assessment characteristics. No studies required exclusion due to extraction errors.

In all extraction rounds, divergent extraction decisions were discussed and resolved, if necessary, by involving additional independent researchers. Missing data, e.g., means and frequencies, were calculated from the information provided in the included publications. No other types of missing data emerged in the sample. Therefore, contacting authors was not necessary.

### 2.6. Risk of Bias Assessment

A critical appraisal of the included sources of evidence was conducted using the Cochrane Risk of Bias 2 (ROB 2) tool, with supplements for cross-over and cluster RCTs [24,25,26] carried out by two independent researchers working in pairs. The tool is a widely recognized and validated instrument for a structured bias assessment specifically designed for randomized studies. The assessment includes five key domains: bias arising from the randomization process, bias due to deviations from intended interventions, bias due to missing outcome data, bias in outcome measurement, and bias in the selection of reported results.

Although critical appraisal is not a standard component of a scoping review, we included this to provide readers with additional context regarding the robustness of the available evidence. As the primary purpose of this scoping review was to map and characterize the available literature, rather than to critically appraise intervention effectiveness, we did not conduct outcome-specific risk-of-bias assessments. We assessed the included studies at the publication level by applying the RoB 2 tool to the primary outcome reported in each publication at the main post-intervention time point. Each publication was assessed separately, even when reporting on the same intervention, because they reported on different outcomes, follow-up periods, and/or analyses, as these factors could influence the risk-of-bias judgment.

### 2.7. Data Analysis and Synthesis

The analysis described the characteristics of the included studies using counts, frequencies, ranges, and narrative synthesis. Interventions were grouped according to diagnosis, setting, delivery mode, professional involvement, intervention components, duration, intensity, comparators, and reported outcomes. Results were summarized using text, tables and figures. The review was reported according to the PRISMA Extension for Scoping Reviews (PRISMA-ScR) guidelines (see Supplementary Materials for the completed PRISMA-ScR checklist) [27].

## 3. Results

**Figure 1:**
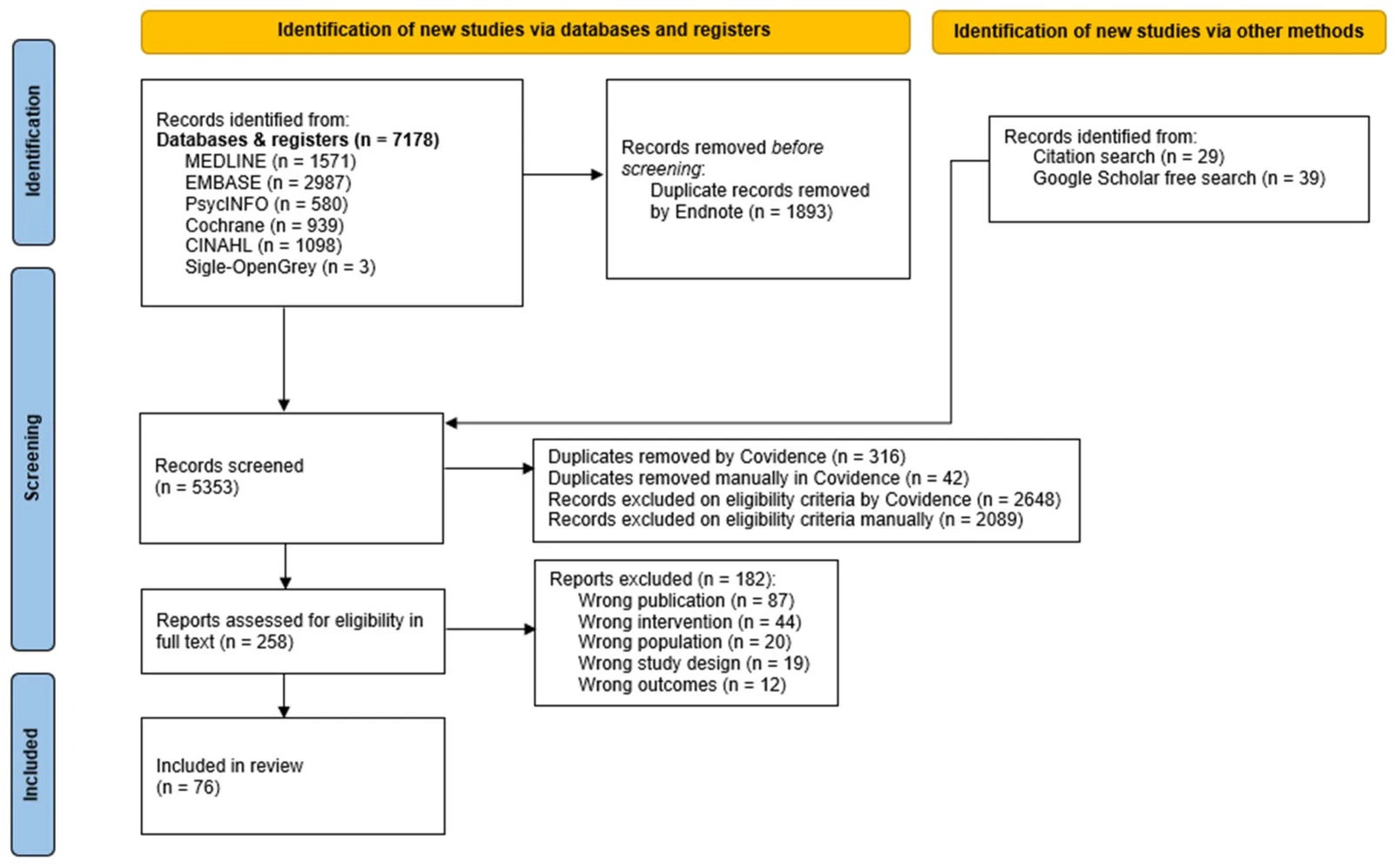
Study flow diagram.

In total, 7246 items were identified through database searching (n = 7178), free web search (n = 39), and citation searching (n = 29) and imported into Endnote, which automatically removed 1893 duplicates. The remaining 5353 records were screened using Covidence software, where an additional 358 duplicates and 4737 records that did not meet the inclusion and exclusion criteria were excluded. Of the 258 relevant publications assessed in full text, 182 were excluded for predefined reasons, resulting in 76 publications eligible for inclusion and data extraction (see Figure 1, PRISMA flow diagram [28]). The 76 included publications reported on 71 interventions spanning 1985–2025 (see Table 2).

In the following, we present the results at the intervention level, consistently distinguishing between publications (n = 76) and trials/interventions (n = 71) and explicitly using the relevant denominator. To prevent double counting, we handled multiple publications arising from the same trial reporting on the same study population as a single intervention, e.g., Ellis et al. 2016, Naar et al. 2014, and Naar-King et al. [35,36,37]; Weinstein et al. 2021 and Martin et al. 2021 [47,48]; Nansel et al. 2007 and Nansel et al. 2009 [95,96]; or Ellis et al. 2005 and Ellis et al. 2008 [99,100]. Studies describing further development of an intervention concept in different populations were considered separate interventions, e.g., Clark et al. 1986, Clark et al. 2004, and Clark et al. 2010 [62,63,64] or Cicutto et al. 2005 and Cicutto et al. 2013 [60,61].

### 3.1. Origin and Design

In total, twelve countries were represented in the sample. Most of the 71 interventions were conducted in the United States (n = 45/71, 63%). The remaining interventions were conducted in Canada (n = 7), the United Kingdom (n = 6), China (n = 3), Australia and Sweden (n = 2 each), and Argentina, Italy, New Zealand, Norway, the Netherlands, and Turkey (n = 1 each). Study designs included full-scale RCTs, including parallel-group (n = 44/71, 62%), cluster randomized (n = 16/71, 23%), and cross-over trials (n = 1/71, 1%). In addition, 10 randomized feasibility or pilot trials were identified, including nine parallel-group studies (13%) and one cluster-randomized study (1%).

### 3.2. Study Populations

The total sample of 71 interventions included 16,342 children and/or adolescents (median sample size n = 157; IQR 81–303; range 12–1316). The median of the reported mean ages across n = 65 interventions was 9 (range 0–19) years. As n = 6 interventions reported approximate age range or the school grades of the participants, these data could not be converted into a comparable age measure and were therefore not included in the age summary. Although eligibility was restricted to children and adolescents aged 0 to 17 years, some included interventions (n = 8/71, 11%) enrolled several participants aged 18–19 years. These interventions were retained as the study population was predominantly within the eligible age range. Girls represented 27–67% of the total study population. The included interventions primarily targeted asthma (n = 48/71, 68%), followed by T1D (n = 21/71, 29%) and JIA (n = 2/71, 3%). One study had a mixed T1D and type 2 diabetes (T2D) population [86]. From this study, only data on T1D were extracted.

### 3.3. Intervention Characteristics

Intervention target population: The 71 interventions targeted children and/or adolescents together with their parents and assessed outcomes in the child and adolescent. However, several trials also included parental outcomes among their primary endpoints [49,63,77,81,92,102]. No siblings participated in the included interventions.

**Figure 2:**
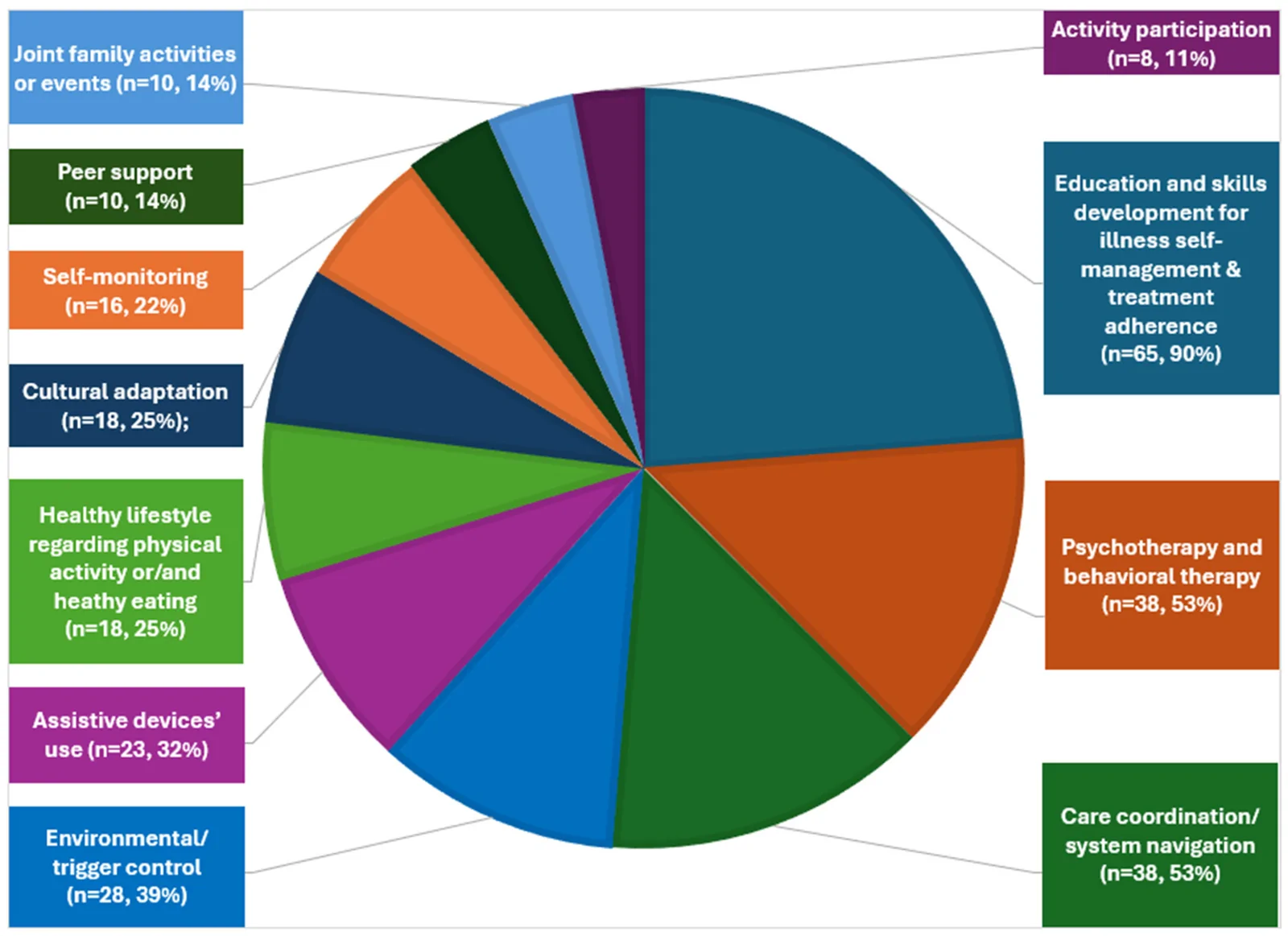
Intervention components. Note. Counts represent the number of times each component was reported across the sample and are non-mutually exclusive. Because most interventions were multicomponent, the sum of component counts exceeds the total number of included interventions (n = 71).

Intervention components: Education, psychosocial and behavioral interventions, as well as care coordination were the most frequently used intervention components. Assistive devices, environmental and trigger control, lifestyle-related content, cultural adaptation, and self-monitoring were used less frequently. In contrast, peer support, shared family activities, and activity participations were included in relatively few interventions (Figure 2).

Intervention duration: Planned active treatments in most interventions (n = 45/71, 63%) varied up to 6 months (1–3 months, n = 26/71; and 4–6 months, n = 19/71). Two trials with an active intervention phase of 1–3 months were transferred to the category of 4–6-month-long interventions [30,74]. This is because the planned 8-week intervention in Brown et al. 2002 [30] was prolonged in several families up to 6 months, and the intervention in Tieffenberg et al. 2000 [74] offered reinforcements at 2–6 months after the 5-week intervention. Longer active intervention periods ranged from 7 to 9 months (n = 3/71) to 10–12 months (n = 12/71), or sometimes up to 2 years (n = 10) or more (n = 1). Interventions spanning a school year (unspecified number of months) were categorized as 10–12 months long, even when the reported intervention period was slightly shorter. For example, Levy et al. 2006 [71] reported a school year duration of approximately eight months. Accordingly, one school term duration in MeGhan et al. 2003 [73] was categorized as a 4–6-month intervention. Post-intervention follow-up periods, usually with few contacts in person, by phone or e-mail with the interventionists, ranged from 0 to 12 months, although several studies reported follow-up periods exceeding 1 year and up to 2 years [47,48,64,70,95,96,97].

Intervention intensity and dose: There were examples of high-intensity interventions, e.g., Multimodal Systemic Therapy (MST) with mean 27–49 therapist contacts and family engagement 2–3 times weekly [35,36,37,85,86,99,100]. Brief treatments contained, e.g., 2–4 home visits delivered by community health workers (CHWs) [31,45], or 6 school sessions, as in the Roaring Adventures of Puff (RAP) school-based self-management asthma program [61,73,79], or Open Airways asthma interventions [62,64].

Mode of the delivery: Most interventions (n = 59/71, 83%) were delivered in person. Overall, 53 of 71 interventions (75%) were delivered exclusively in community settings (n = 20/71) or combined with home (n = 16/71), clinic (n = 7/71), telehealth (n = 6/71), or both home and clinic settings (n = 4/71). Of the community-only interventions, 12/20 were delivered in school settings, and 2/20 in physical activity facilities. Of the remaining 18/71 interventions (25%), 13/18 interventions were delivered exclusively at home (including four using telehealth), 4/18 interventions combined home and clinic settings (including home–clinic and home–clinic-telehealth delivery, in two interventions each), and 1/18 intervention was delivered exclusively in a clinic setting [93]. Although delivered in clinical locations, the latter was provided by non-medical care ambassadors and therefore considered eligible. Personal contact had frequent phone, text, or e-mail adjuncts for follow-up. Half of the digital interventions that applied home and community usage (n = 6 of 12/71, in total) were self-guided, including four with personal follow-up contacts by phone. Health professionals from various disciplines, where nurses were the largest group, delivered the experimental contents as primarily deliverers in 44/71 (62%) interventions (Table 3). Other professionals, e.g., trained staff, researchers, schoolteachers, social workers, or unspecified, were primary deliverers in 30 (42%) interventions. Lay persons, e.g., community health workers or peers, delivered interventions in 17/71 (24%) interventions.

### 3.4. Comparators

Most interventions compared the experimental contents with usual/standard care, e.g., medication control, education materials, or consultation—sometimes including in-home visits but no explicit community-based approach (n = 42/71, 59%). Other active treatments appeared less frequently as comparators (n = 22/71, 31%). In total, 11 of 71 (15%) interventions used waitlists/no intervention as comparison, and one intervention (1%) [72] used their own controls.

### 3.5. Outcomes

**Figure 3:**
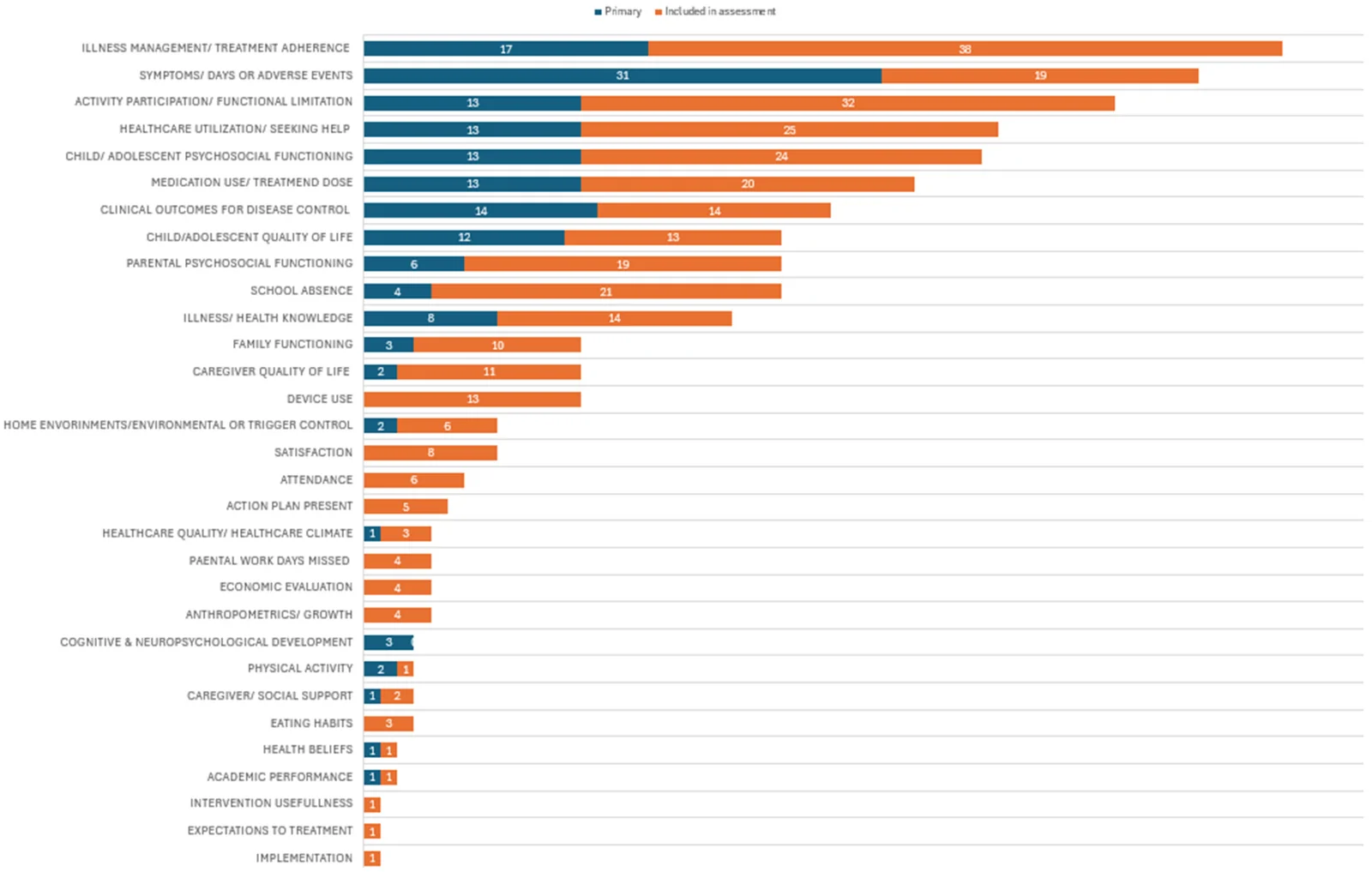
Outcomes assessed in included publications. Note. Numbers refer to the number of times an outcome was met in the sample.

The 71 interventions most frequently assessed the following as their primary outcomes: symptoms or adverse events, e.g., symptom-free days or days with symptom aggravation (n = 31/71, 44%); illness management, e.g., individual or family coping with the disease (n = 17/71, 24%); clinical indicators of disease control, e.g., blood glucose or lung function (n = 14/71, 20%); activity participation (n = 13/71, 18%); healthcare utilization, e.g., emergency department visits or hospitalizations (n = 13/71, 18%); psychosocial functioning of the child or adolescent (n = 13/71, 18%); medication use or treatment intensity (n = 13/71, 18%); and quality of life of the child or adolescent (n = 12/71, 17%) (see Figure 3). Similar patterns were also seen in the choice of secondary outcomes, with an addition of school absence (n = 21/71, 30%) and parental psychosocial functioning (n = 19/71, 27%) among the most frequently assessed outcomes.

Outcomes such as device use, satisfaction, attendance, action plan present, parental workdays missed, economic evaluation, anthropometrics/growth, eating habits, intervention usefulness, expectations of treatment, or implementation were only assessed as secondary outcomes in relatively few interventions.

### 3.6. Family- and Community-Centered Care

**Figure 4:**
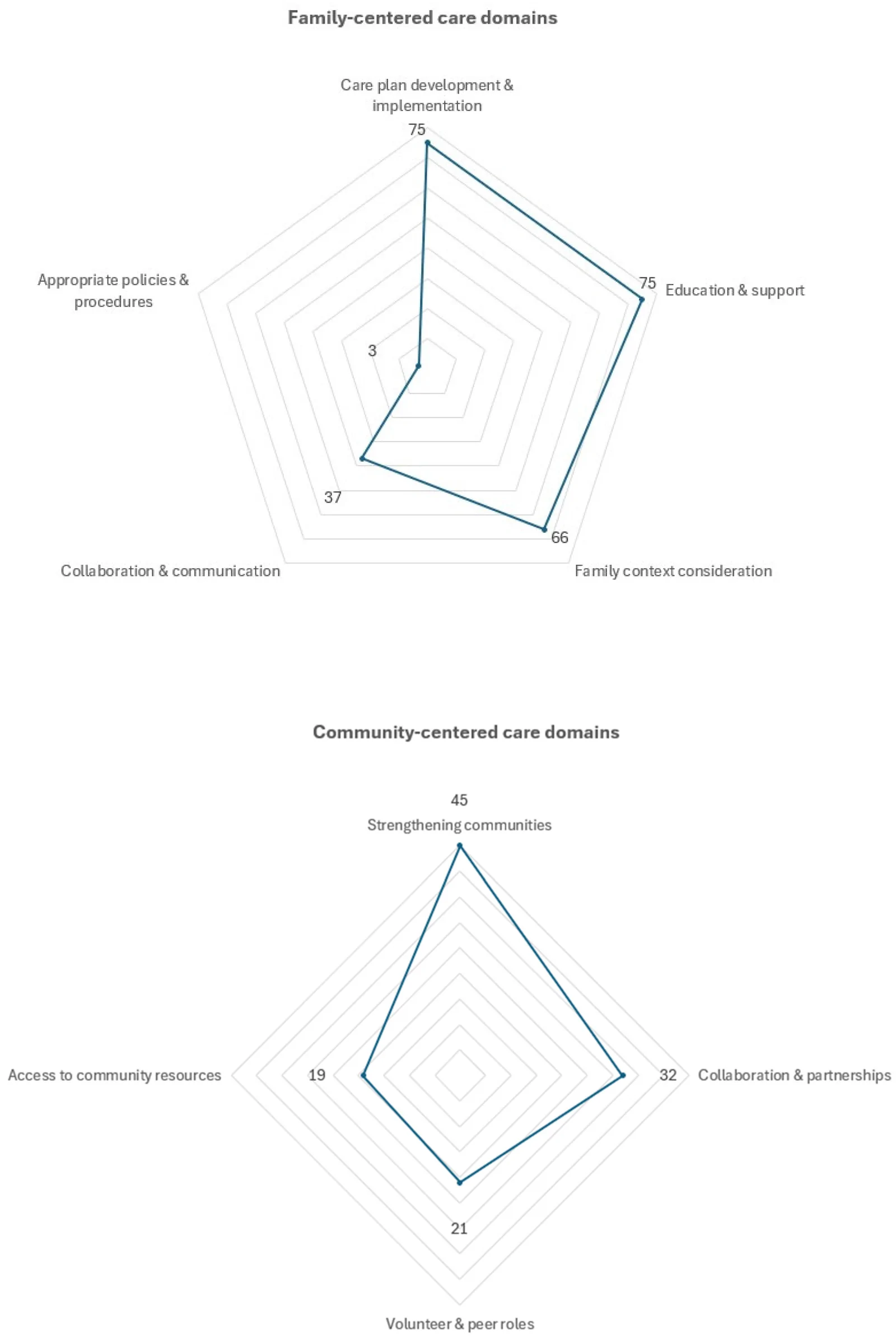
Sample trials’ coverage across family- and community-centered care (images created by the authors for this study, based on concepts presented in Kokorelias et al. and South et al. [20,22]). Note. As the included interventions (n = 71) covered several domains, the counts are non-mutually exclusive, and the total number of components exceeds the total number of interventions in the sample.

According to the framework proposed by Kokorelias et al. [22], most of the 71 interventions included in this review incorporated individualized care, problem-solving, and shared decision-making (i.e., primarily corresponding to the care plan development and implementation domain) or illness management, education, and support (i.e., the education and support domain) within family-centered care (see Figure 4). Many interventions also included components corresponding to the collaboration and communication domain, particularly those focused on care coordination and communication between sectors. A total of 3 of 71 (4%) interventions [40,61,74] incorporated policymaking corresponding to the appropriate policies and procedures domain. However, these interventions reflected only micro-level policy development within school settings, with no examples of policymaking initiatives to support family-centered care on community or national levels.

Asthma interventions most comprehensively spanned all five family-centered care domains, likely reflecting the need for disease management across multiple settings, including the home, school, and healthcare system. Several asthma programs also incorporated elements of the appropriate policies and procedures domain more actively, such as school asthma protocols, which were less evident in other conditions.

Interventions targeting T1D placed greater emphasis on family context, explicitly addressing family routines, parental roles, conflict, emotional burden, and the transition towards self-management and autonomy. Policy- and procedure-related components were less common and were typically embedded within clinical services rather than implemented at a broader systems level.

Interventions targeting JIA were predominantly digital or coaching-based and typically focused on education and support, self-management, coping skills, and caregiver guidance. Care planning was generally limited to individual goal setting rather than coordinated implementation across healthcare, family, and community systems. Components related to collaboration, consideration of the family context, and policy development were comparatively underrepresented.

According to the domains in community-centered approaches for health proposed by South et al. [20], this review showed that many sample trials contributed to the domain strengthening communities setting, with an emphasis on collective efficacy and social connectedness, e.g., as in school-based and culturally adapted interventions. The volunteer and peer roles domain was strongly connected to the involvement of community health workers and peers in the interventions (n = 21/71, 30%). Many interventions explicitly incorporated structured collaboration or/and partnerships between healthcare providers and community-centered services (e.g., in multisystemic interventions, comprehensive school-based asthma programs, and integrated community health worker models). These interventions aligned with the collaborations and partnerships domain (n = 32/71, 45%). In addition, some interventions (n = 19/71, 27%) included components aimed at facilitating access to community resources, such as resource navigation, material support, environmental remediation, or linkage to social services, corresponding to the access to community resources domain. However, these components were typically supplementary to the intervention’s primary focus rather than serving as its central element.

Asthma interventions most frequently spanned all four community-centered care domains, reflecting the condition’s strong alignment with school-based programs, CHW models, environmental remediation, and cross-sector collaboration. T1D interventions commonly emphasized family strengthening and peer or volunteer coaching, with fewer interventions explicitly focused on material or environmental resource access. JIA interventions were primarily digital or home-based, engaging peer or coach roles but rarely incorporating broader community partnerships or resource navigation.

### 3.7. Methodological Quality Evaluation

**Figure 5:**
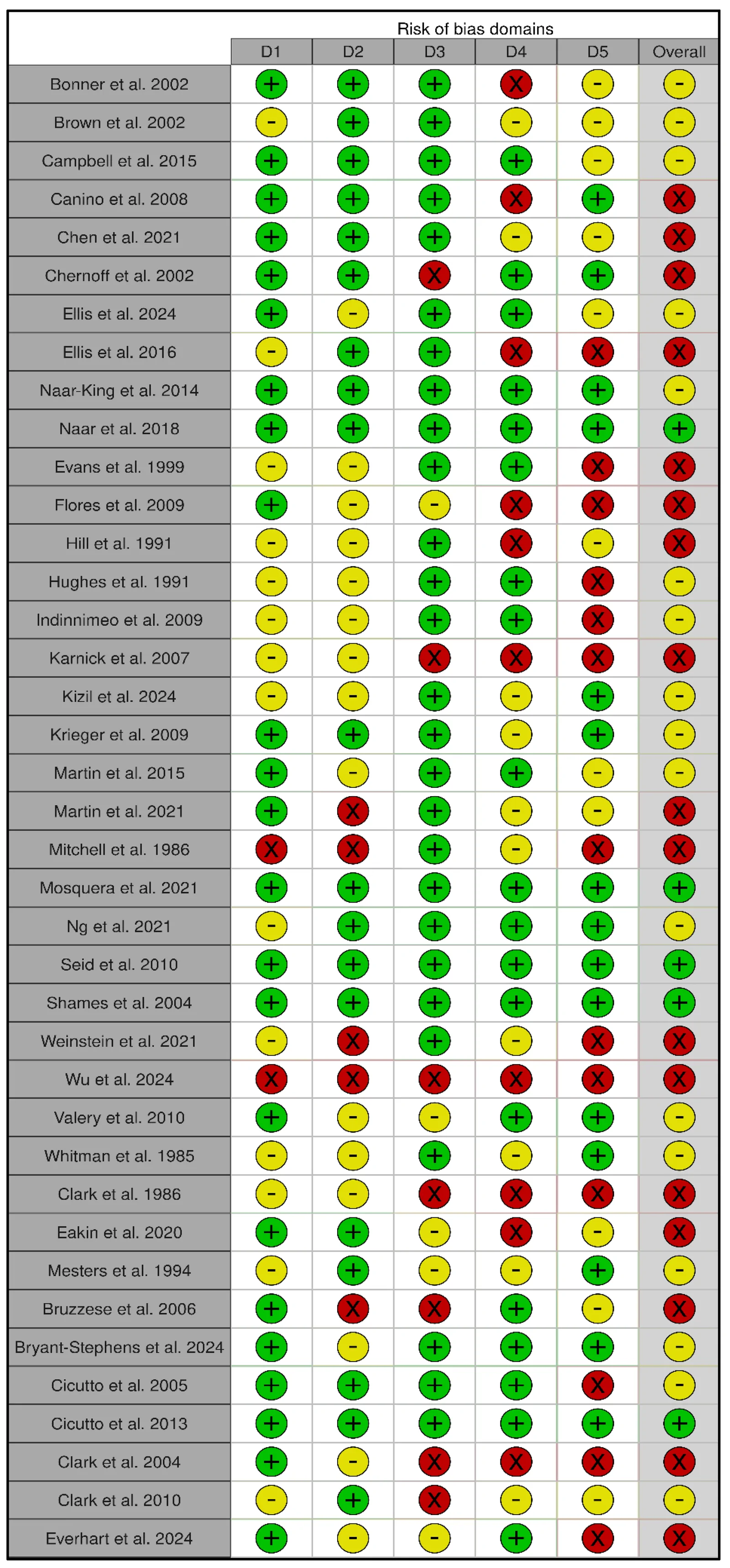

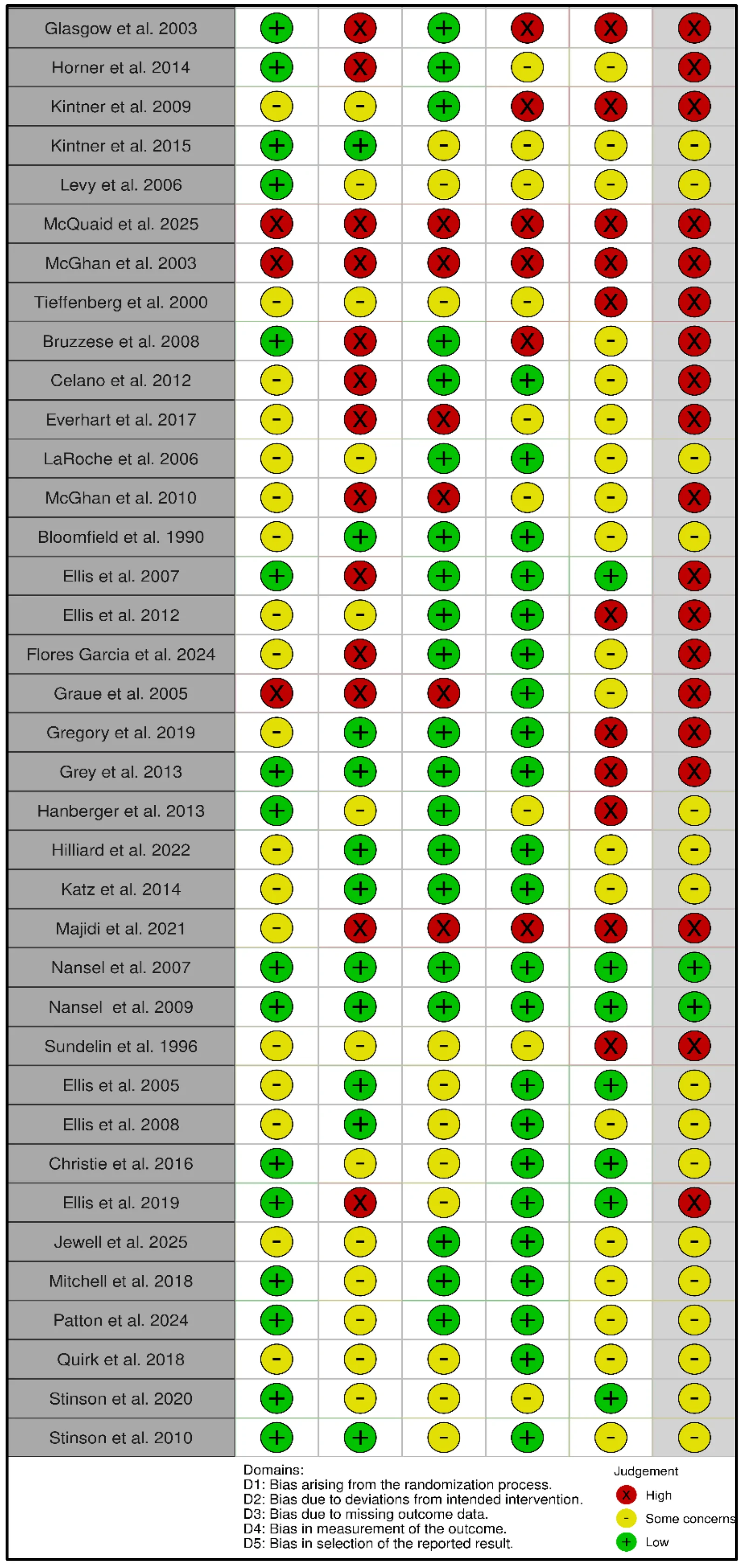
Methodological quality evaluation using Rob2 tool [23,24]. Note. JIA, juvenile idiopathic arthritis; T1D, type 1 diabetes [29,30,31,32,33,34,35,36,37,38,39,40,41,42,43,44,45,46,47,48,49,50,51,52,53,54,55,56,57,58,59,60,61,62,63,64,65,66,67,68,69,70,71,72,73,74,75,76,77,78,79,80,81,82,83,84,85,86,87,88,89,90,91,92,93,94,95,96,97,98,99,100,101,102,103,104].

Risk of bias was assessed at the publication level instead of intervention level, as some interventions were reported across multiple publications, to ensure that all available information relevant to risk of bias in the included publications (n = 76) was considered. In accordance with RoB 2 guidance [24,25], publications with at least one domain rated as high risk were classified as having overall high risk of bias. Overall risk of bias was judged as high (n = 36/76, 47%) or some concerns (n = 33/76, 43%), with few studies at low risk (n = 7/76, 9%) (Figure 5). Concerns most frequently arose from measurement of outcomes and selective reporting, while randomization procedures and missing outcome data were generally better addressed. These limitations should be considered when interpreting the results.

Randomization process (D1) was most frequently judged at low risk or as having some concerns because allocation concealment was frequently unclear, particularly in older studies. A small number of trials were judged at high risk due to an inadequate or insufficiently reported randomization approach. Lack of blinding and limited information on whether deviations from assigned interventions were balanced between groups was often the reasons for some concerns or high risk ratings regarding bias due to deviations from intended interventions (D2). For bias due to missing outcome data (D3), many studies had acceptable levels of follow-up or appropriate handling of missing data and were judged at low risk of bias, while substantial attrition or insufficient reporting of reasons for missing data resulted in high risk judgment in several studies. Bias in measurement of the outcome (D4) was a common source of concern because many trials were relying on subjective outcomes, which was judged at high risk of bias, particularly when combined with a lack of blinding typical for the sample interventions. Limited availability of prespecified protocols or analysis plans was usually the reason for some concerns-judgment regarding bias in selection of the reported results (D5), while several studies were judged as having high risk of bias due to indications of selective outcome reporting.

## 4. Discussion

This scoping review provides an overview of the characteristics of 71 trials reported in 76 publications on family- and community-centered healthcare interventions for children and adolescents with asthma, T1D, and JIA, including intervention components, duration, intensity and dose, mode of delivery, comparators, and reported outcomes. The review demonstrates that family-centered, community-based interventions for children and adolescents with asthma, T1D, and JIA are predominantly short-term (≤6 months), multicomponent interventions that emphasize education, psychosocial support, and care coordination. These interventions are typically delivered by healthcare professionals in community settings beyond traditional clinical environments. Symptom burden and illness management were the most frequently assessed outcomes, while usual care served as the most common comparator.

This scoping review showed that 68% of the 71 included interventions focused on asthma, indicating substantial variation in diagnosis coverage, revealing a scarcity of family- and community-centered approaches in T1D, and especially in JIA. The distribution of studies across diagnostic groups in this review, with T1D and JIA accounting for 29% and 3% of the sample, may reflect the predominantly clinic-based nature of care for these conditions and support previous observations that family-centered, community-based approaches remain less developed in conditions traditionally managed within specialist healthcare services [17]. Consistent with the observations of South et al. [20], our findings suggest that conditions with strong environmental and social determinants, such as asthma, are more likely to be addressed through whole-system approaches. In contrast, conditions predominantly managed within specialist care, such as T1D and JIA, are more often associated with targeted participatory strategies rather than broader system-oriented interventions. The underrepresentation of non-medical professionals, lay persons (CHWs), and peers in the interventions identified in this review may constrain the reach of family- and community-centered approaches. Increasing their involvement could support the delivery of health-promoting initiatives beyond clinical settings and across diagnostic categories.

Family- and community-centered interventions included in this review were predominantly multicomponent and delivered outside traditional healthcare settings. The recurrence of these components suggests that they constitute core building blocks in current intervention development, which is in line with previous evidence [15,16]. Although the present review did not assess comparative intervention effectiveness, previous systematic reviews have reported reductions in emergency department use and symptom burden associated with multicomponent community-based asthma interventions [105,106]. Frequent use of care coordination may reflect international conceptualization of family-centered care as partnership-oriented and context-sensitive [17,107], highlighting the importance of interdisciplinary, shared efforts and real-life adaptation in family- and community-centered interventions.

Given that most interventions are multicomponent and often compared with heterogeneous forms of usual care, it can be difficult to understand how educational, psychosocial, care-coordination, and other intervention elements represented within the sample trials contribute to the observed effects. Thus, the present mapping cannot determine which individual components are associated with greater effectiveness, or which components may be most relevant in different contexts and settings. The lack of understanding points to the need for more robust research in the field. This gap can be addressed in future family- and community-centered trials by adopting frameworks such as the context-trial driver model proposed by Turner et al. [108] or the multi-method usual-care characterization approach proposed by Yorganci et al. [109].

The primary outcomes of the included trials may not fully reflect the outcomes that are most important for children and families. A recent systematic review showed that pediatric core outcomes frequently extended beyond biomedical measures to include functioning, quality of life, and patient-reported outcomes, though substantial heterogeneity exists across conditions [110]. Our findings are in line with a recent review demonstrating that while physical and social participation is valued by families, it is rarely the primary target of family-centered care interventions [111]. Additionally, outcomes such as school absenteeism, parental work absenteeism, and medication use were assessed relatively infrequently in the included studies. The increasing rates of school absenteeism among Danish children and adolescents [112] and the high levels of stress among adults of parenting age [9,10] indicate that outcomes such as school attendance and parental work participation may be important indicators of intervention effectiveness [110]. Their infrequence suggests that important benefits of family- and community-centered interventions may currently be overlooked.

By addressing challenges in everyday life participation and evaluating outcomes with broader societal relevance through both objective and subjective measures, the benefits of family- and community-centered interventions can be more fully captured at both the individual and societal levels. Although this will probably raise the research costs, such measures and user voices are essential for capturing the full value of these interventions.

The findings of this review also support the observation of Kokorelias et al. that family-centered care is most commonly operationalized through educational and interpersonal approaches, while organizational policies and procedures remain the least developed components [22]. Only a few interventions included the development or implementation of policies and procedures beyond the individual or family level, and these were primarily confined to school settings. This finding suggests that family- and community-centered interventions primarily targeted individual and interpersonal determinants of behavior, while paying limited attention to broader system-level influences. According to the Behaviour Change Wheel, policy categories, such as guidelines, legislation, environmental and social planning, are key mechanisms for supporting sustainable behavior change [113]. Thus, the absence of community- and national-level policy initiatives identified in this review may represent a missed opportunity to support potentially broader and more sustainable changes beyond the individual and family level.

### 4.1. Strengths and Limitations

The strengths of this study include a rigorous search strategy across multiple databases, inclusion of the gray literature, and comprehensive quality assurance procedures, including independent screening and data extraction by multiple reviewers. These measures helped minimize the risk of selection and extraction bias and enhanced the reliability of the review process.

However, several limitations should be considered when interpreting the findings of this study. Regarding the literature search and data extraction, not capturing potentially relevant studies published in languages other than English may have excluded relevant evidence and systematically affected the findings in this review. OpenGrey consulted in this review has not been updated since 2020 and therefore could not identify recently completed, ongoing, or newly unpublished studies. An automated tool used during the initial stage of data extraction may have introduced errors despite subsequent verification procedures. However, the approach used in this reduced the risk of errors arising from translation and facilitated a standardized review process across studies, with independent researchers involved at all stages of screening, data extraction, and quality assessment. Nevertheless, future reviews in this field should include searches of clinical trial registries to improve the identification of ongoing, completed but unpublished, and recently registered studies.

Several limitations also refer to transferability and generalizability of the results. For example, eligible asthma interventions predominantly originated from the United States. Together with the review’s focus on three diagnostic groups, this may have limited the generalizability and transferability of findings across a broader scope of pediatric chronic conditions and settings. Additionally, a small proportion of participants in the interventions included fell outside the predefined eligible age range for this study. Although limited to a few interventions, this may nevertheless limit the generalizability of the findings to children and adolescents aged 0 to 17 years. Moreover, the included studies spanned several decades, during which the family- and community-centered approaches may have evolved, resulting in substantial variation in the definitions, standards, or practices of family- and community-centered care represented in the sample.

Additionally, the exclusion of qualitative and observational studies targeting patient perspectives may have limited the study’s scope and the insights obtained through data analysis. However, limiting our scope to RCTs helped us conduct focused research.

Assessing risk of bias at the publication level in this scoping review, rather than conducting comprehensive outcome-specific assessments, enabled evaluation of all included publications. However, this approach did not capture risk of bias across different outcomes, time points, and analyses in the sample. Therefore, the assessment results should be interpreted as an overall indicator of the methodological quality of the included evidence. Future systematic reviews may address this limitation by conducting comprehensive outcome-specific risk-of-bias assessments and providing a more nuanced understanding of the intervention effects.

### 4.2. Implications

This scoping review highlights the need to advance the evidence base and implementation of family- and community-centered care.

In practice, efforts should focus on the following:

- Strengthening organizational and policy-level approaches by addressing organizational structures, service integration, and healthcare policies;
- Involving a wider range of stakeholders, such as non-medical professionals, community health workers, and peers to support traditional healthcare services.

Further research should consider the following:

- Developing robust diagnosis-inclusive family- and community-centered interventions applicable to diverse patient populations;
- Addressing challenges related to participation in everyday life and capturing the interventions’ impact on both the individual and societal levels.

## 5. Conclusions

This scoping review found that most included family- and community-centered interventions were predominantly short-term (≤6 months), delivered by healthcare professionals in community settings, commonly combined educational, psychosocial, and care-coordination components, and compared the experimental contents with usual care. Symptom management and disease control were the most frequent primary outcomes. Inclusion of populations with mixed diagnoses, peer-support, shared family activities, or delivery by non-professionals were uncommon.

This study also revealed an uneven diagnostic focus, heterogeneity in intervention and outcome assessment methods, and limited attention to policymaking initiatives in family- and community-centered care. Future research should prioritize high-quality trials that broaden diagnostic coverage, incorporate underrepresented intervention components, and evaluate innovative delivery models to strengthen the evidence base for family- and community-centered healthcare for children and adolescents with chronic somatic conditions.

## Figures and Tables

**Table 1:** Inclusion and exclusion criteria.

Item	Inclusion Criteria	Exclusion Criteria
Participants	Children and adolescents aged 0–17 years diagnosed with asthma, T1D, or JIA, alone or in combination with other chronic somatic diseases, and their families	Children and adolescents with (a) mental conditions or psychiatric diseases (i.e., non-somatic), or (b) combined diagnoses including non-somatic diseases or developmental disorders, or (c) life-threatening diagnoses, e.g., cancers; palliative care interventions; perinatal or neonatal interventions
Concept	Any single or multicomponent family-centered intervention promoting health behavior and well-being (e.g., improving physical activity and eating habits) *	Studies on interventions that deliver medical treatments or surgery, or environmental modifications; interventions focused on parents as primarily recipients
Context	Interventions delivered in or through community settings, actors, or systems beyond specialized clinical care *	Interventions not involving communities
Study design	RCT	Non-randomized trials, observational studies (i.e., cohort, case–control or cross-sectional), qualitative studies, and review articles)
Timespan	No limits	
Language	English	

* Note. Main components of community- and family-centered care are conceptually inspired by the work of Kokorelias et al. and South et al. [20,22]; no copyright permission required.

**Table 2:** Included interventions.

Study	Sample n	Age Mean/Range	Females	Settings	Community Anchorage	Intervention Components	Intervention Duration	Intervention Intensity/Dose	Intervention: Mode of the Delivery	Comparator	Outcome Assessment *
**Asthma**											
Bonner et al. (2002), USA [29]	Total n = 119; IG n = 56; CG n = 63	10 (4–19)	43%	University hospital	Community environments and community homes	Family Coordinator: asthma self-regulation education, homework, trigger reduction, monitoring, and care coordination	3 mo	12–14 h/wk of phone support (avg. 30 min. with each phone contact); accompanied 1–2 doctor visits; 2 home visits	Individual and family-oriented; group workshops, one-to-one phone coaching, home visits, clinic visit accompaniment (in-person); culturally tailored	Usual care	Asthma symptom persistence/severity *; Activity restriction; Knowledge about asthma; Western biomedical beliefs; Non-Western health beliefs; Self-efficacy for managing asthma; Pharmacotherapy; Family’s adherence; Prophylactic bronchodilator use; Symptom persistence; Activity restrictions
Brown et al. (2002), USA [30]	Total n = 95; IG n = 49; CG n = 46	4 (1–7)	45%	Community clinics	Community homes	Wee Wheezers: developmentally tailored asthma education with caregiver–child activities, materials, and supervised home-visitor delivery	8 wks (extended for median 10 wks; range 1.9–24.4 wks for most families)	8 sessions of 90 min. (1 session/wk)	Individual and family-oriented; in-home nurse visits; caregiver–child joint participation; interactive demonstrations; homework; home inspections; individualized family-focused instruction	Usual care	Asthma morbidity*; Symptom-free days; Acute asthma visits; Caregiver QoL; Adherence to daily medical regimen; Medication during early upper respiratory infection signs; Recognition of early warning signs; Symptom management and prevention practices; Environmental exposures; Child participation in medication tasks; Child participation in prevention and treatment tasks
Campbell et al. (2015), USA [31]	Total randomized n = 373; IG n = 182; CG n = 191	8 (3–17)	39%	Low- income communities	Community homes	CHW home-visit program: motivational interviewing-based education, device coaching, environmental remediation, and provider coordination	3.5 mo with 12 mo follow-up	4 sessions (appx. 1 session/mo)	Family-oriented; in-home with supplementary phone or e-mail support; culturally tailored	Usual care	Symptom-free days over prior 2 weeks*; Nights with symptoms; Days with activity limitation; Days using rescue medication; Asthma episodes; Asthma control level; Urgent asthma-related health services; Economic evaluation
Canino et al. (2008), Puerto Rico/USA [32]	Total n = 221; IG n = 110; CG n = 111	7 (5–12)	37%	Low- income communities	Community homes	CALMA: 8 tailored asthma education modules with manuals, counseling, and structured fidelity monitoring	4 mo	2 sessions; 8 modules (mean 18.4 days between visit 1 and 2); phone calls	Family-oriented; in-home counseling by trained asthma counselors; culturally tailored	Usual care and flyers	Symptom-free days*; Symptom-free nights; Asthma control; Activity limitations; Emergency department visits and hospitalizations; Medication use; Caregiver QoL; Caregiver asthma knowledge; Family empowerment; Caregiver self-efficacy
Chen et al. (2021), China [33]	Total n = 120; IG n = 60; CG n = 60	4 (3–7)	35%	Tertiary hospital	Community homes	Hierarchical education tailored by child functioning level and home visits	6 mo with 3 and 6 mo follow-up	Varied after stratification; intermediate level added home visits every 2 mo; enhanced level added weekly data analysis	Individual and family-oriented; nurse-led program; in-person and digital delivery via WeChat; phone support and home visits	Other treatment (no hierarchical approach)	Acute asthma attacks & emergency visits & hospitalizations*; Asthma Control Test; PEF daily variation rate; Pulmonary function; Serum inflammatory markers; Medication adherence; QoL
Ellis et al. (2024), USA [34]	Total randomized n = 59 (analyzed n = 57; IG n = 28; CG n = 29	15 (12–16)	51%	Urban pediatric hospital	Community homes	Reach for Control (RFC): multisystem family CBT-based intervention; individualized plans and coordination across family, school, and healthcare	6 mo; Phase 1 wks 1–4; Phase 2 wks 5–20; Phase 3 wks 21–24	Planned dose 24 sessions (1 session/wk); observed mean sessions 4.29 ± 4.22 overall	Individual and family-oriented; trained CHW delivery; in-person/ in-home	Other treatment: home-based with a lower intensity than RFC	Asthma-related emergency department visits*; FAMSS; Caregiver QoL; Adolescent QoL; Pulmonary function; Asthma control; Socioeconomic status; Intervention satisfaction
Naar-King et al. (2014), USA [35]; Ellis et al. (2016), USA [36]; Naar et al. (2018), USA [37]	Total randomized n = 170 (analyzed n = 167); IG n = 84; CG n = 83	13 (12–16)	39%	Urban asthma clinic or hospital	Community schools and community homes	Multisystemic therapy healthcare: multisystem therapy targeting skills, family routines, school coordination, and system barriers	6 mo; mean 5.14 mo with 7 and 12 mo follow-up	Mean n = 27 sessions (SD 12; range 4–62 sessions)	Individual and family-oriented; in-home family sessions; contacts with schools and medical providers	Family Support 45 min./wk of in-home empathic support and advise	Asthma knowledge*; Controller device use skills; Illness management follow-up: pulmonary function*; Medication adherence; Daily Phone Diary; Symptom response
Evans et al. (1999), USA [38]	Total n = 1033; IG n = 515; CG n = 518	8 (5–11)	36%	Community asthma clinics	Community neighborhoods and community homes	National Cooperative Inner-City Asthma Study: Asthma counselor program with individualized education, group sessions, environmental controls, and ongoing follow-up	24 mo	Number and length of contacts tailored to asthma profile; phone calls every 2nd mo	Individual and family-oriented; in-person; in-group (families and peers); phone calls and environmental home supports	Usual care (a blank asthma plan, spacer, peak flow meter, and NHLBI guidelines)	Asthma symptom days*; Hospitalizations and unscheduled visits
Flores et al. (2009), USA [39]	Total n = 220; IG n = 112; CG n = 108	7 (2–18)	43.6%	Community hospitals, community centers/churches and private homes	Out-of- hospital support involving trained community members	Parent mentors: peer-mentor (trained experienced parents) support with home visits, calls, education, and resource navigation	12 mo	12 monthly calls and meetings (n = 57 in total across venues); 2 home visits at baseline and 6 mo; mentors available 24/7	Family and peer-oriented; in-person; in-group (families and peers); peer-led with nurse oversight; community group meetings; home visits; phone calls; culturally tailored	Usual care	Monthly exacerbations/symptom episodes * (wheezing; coughing; difficulty breathing; rapid breathing; chest tightness); Emergency department visits; Hospitalizations; Healthcare use and costs; Missed school days and missed parental workdays; Pediatric QoL; Pediatric Asthma Caregiver’s QoL; Parent asthma management self-efficacy; Asthma Satisfaction Survey
Hill et al. (1991), UK [40]	Total n = 451; IG n = 228; CG n = 223	NI (5–10)	43%	Community primary schools	Community school-based support system	GP consultations; teacher education; instructions to parents and GPs; inhaler technique checks	1 academic year	Activities coordinated across the academic year	Family and peer-oriented; in-person; in-group (families and peers); nurse-led	Usual care	School absence*; Participation in games and swimming*; School asthma management practices; Child access to inhalers and pre-exercise use (parent and teacher questionnaires); Teacher knowledge and preparedness (post-session test and survey)
Hughes et al. (1991), Canada [41]	Total n = 95; IG n = 47; CG n = 48	10 (6–16)	37%	Community hospital and private homes	Out-of- hospital care added	Comprehensive anticipatory management with regular follow-ups at the hospital with home visits, and coordinated care	12 mo with 12 mo follow-up	3 physician visits/year; 2 home visits by nurse/year	Family and peer-oriented; in-person; in-home; on-phone support; physician and nurse-led	Usual care	Asthma severity*; Anthropometrics: height, weight, blood pressure, airway obstruction, cough, eczema, allergic rhinitis; Smoking and pet exposure documented; Serum theophylline levels (μmol/L or mg/dL) where applicable; Pulmonary function; Hospital admissions and days; Emergency department and office visits; Metered aerosol technique rated as good/fair/poor using predefined criteria
Indinnimeo et al. (2009), Italy [42]	Total n = 123; IG n = 60; CG n = 63	9 (6–14)	42%	Outpatient clinics	Community health centers	Long-term Educational Program for Children with Asthma-Aironet (LEPCA-A): education with interactive discussion on asthma knowledge and self-management	12 mo	3 sessions in total: 2 education sessions of 90 min. at baseline and 2 mo	Family-oriented; in-person group sessions for children and parents at asthma clinics	Usual care	Asthma severity* (asthma attacks, medications, unscheduled family physician or emergency visits, hospitalizations, school absences); Pulmonary function; Parent illness knowledge
Karnick et al. (2007), USA [43]	Total n = 212; IG1 n = 74; IG2 n = 68; IG3 n = 70	6 (1–16)	40%	Hospital emergency department in disadvantaged inner-city community	Out-of- hospital support involving lay health educators	3-arm education model: (1) individualized education; (2) reinforced education; (3) reinforced education plus case management	9 mo Data collected for 12 mo before the study and on monthly follow-up for 9 mo after enrollment/treatment from pulmonologist	Varying intensity add-on to baseline pulmonologist visit: (1) single 20–30 min education session; (2) monthly reinforcement contacts as needed; (3) case management by nurse practitioner avg. of 10.8 h over 9 mo (range 2–78 h) plus reinforced education	Family-oriented; in-person education by trained asthma lay educator; monthly phone follow-ups for data collection and reinforcement; nurse-led case management for IG3 with health educator support	3 active treatments compared	Asthma-related health resource utilization*; Hospital days; emergency department visits; Unscheduled clinic visits); School days missed; Cost–benefit
Kizil et al. (2024), Turkey [44]	Total randomized n = 94 (analyzed n = 92); IG1 n = 31; IG2 n = 31; CG n = 30)	8 (7–10)	39%	Community primary schools	Cross- sectoral support bridging schools and community homes	School-based asthma education with materials, demonstrations and follow-up calls, 2 arms: (1) child–parent; (2) child-only	<3 mo	1 education session (40 min); 1 booklet per participant (parent/child versions); 3 brief monthly calls after education	Family and peer-oriented; in-person; in-group education at school; remote follow-up by phone	Other treatment: CG only targeted children and not parents	Asthma Control Test*; Rescue medication use
Krieger et al. (2009), USA [45]	Total n = 309; IG n = 156; CG n = 153	8 (3–13)	36%	Community public health clinics and private homes in low- income communities	Co-design with user involvement (community advisory board) and CHW- delivery	Nurse education plus CHW support (home assessment and tailored support)	12 mo	Nurse-led structured education with up to 3 follow-up clinic visits; CHW contact with avg. 3.1 follow-up home visits	Family-oriented; in-person clinic education by nurses; in-home visits by CHWs; phone contacts as needed; coordinated communications with providers	Other treatment: Nurse education only	Symptom-free days*; Caregiver QoL; Urgent health services use; Activity limitation days; β-agonist use days; Asthma attacks; Missed school/workdays; Controller daily use; Spacer use; Written action plan; Asthma-control action score; Environmental actions; Self-regulation; Social support; Self-efficacy
Martin et al. (2015), USA [46]	Two cohorts, n = 101: Elementary School Cohort (ESC): Total n = 51; IG n = 26; CG n = 25; High School Cohort (HSC): Total n = 50; IG n = 25; CG n = 25	13 (5–17) ESC: 9 (5–14); HSC: 16 (15–17)	ESC: 39% HSC: 58%	Community clinics	Community neighborhoods and community homes; CHW involvement	CHW home visits: education, self-management coaching, and behavior-change planning	4 mo with 12 mo follow-up	4 home visits	Family-oriented; in-person; in-home CHW education and coaching; mailed newsletters for control; culturally tailored	Other intervention: attention-control newsletters covering identical topics	Medications in the home*; Medication adherence; Inhaler technique; Home trigger score; Asthma control; Depressive symptoms; Life events; Aeroallergen sensitization; Clinical utilization
Martin et al. (2021), USA [47]; Weinstein et al. (2021), USA [48]	Total n = 223; IG (CHW) n = 108; CG (asthma educator) n = 115	9 (5–16)	44%	Community health centers and community homes	Community homes; CHW involvement	Extended CHW program: education, adherence support, and care coordination	12 mo with 6, 12, 24 mo follow-up	10 home visits	Family-oriented; in-person; in-home; culturally tailored	Other intervention: Standard asthma education delivered by certified asthma educator	Asthma Control Test*; Activity limitation days; Depression; Post Traumatic Stress Disorder (PTSD) symptoms; Family chaos; Social support
Mesters et al. (1994), Netherlands [49]	Randomized n = 108; Total n = 81; IG n = 33; CG1 = 30; CG2 n = 18	3 (0–4)	35%	Community health centers and GP	Community GP involvement	Modular asthma manual: structured education with home reading and discussion	6 mo with 1 year follow-up (only for IG)	Avg. of 5 GP-family contacts; community asthma nurse involvement minimal; fidelity varied	Family-oriented; in-person; GP-led; occasional home visits by nurses	CG1: waitlist; CG2: medical outcomes control	Parent’s knowledge*, attitude*, disease management*, anxiety*, asthma symptoms*; GP-reported medical care utilization (nonemergency and emergency visits; hospitalizations); Asthma severity; Episode duration categories; Process outcomes including module completeness; fidelity; cooperation among GPs, nurses, and health center physicians
Mitchell et al. (1986), New Zealand [50]	Total n = 368; European cohort: IG n = 94, CG n = 106; Polynesian cohort: IG n = 84, CG n = 84	6 (2–14)	42% (Europeans), 39% (Polynesians)	Community hospital and community homes	Out-of- hospital care	Monthly nurse home visits: education, inhaler technique, and environmental control guidance	6 mo with 6 and 18 mo follow-up	Max. 6 visits/mo	Family-oriented; in-person; in-home individualized nurse visits	Usual care	Readmissions*; Number of asthma medications*; Severe asthma attacks*; Missed school days*; Treatment location; Illness management*
Mosquera et al. (2021), USA [51]	Total n = 63; IG n = 34; CG n = 29	10 (2–18)	41%	Community clinic	Community schools and community homes	Comprehensive care plus Asthma Intervention Program (AIP): primary care plus monitoring by motivational interviewing, refill monitoring, and home PEF-tracking	6 mo with appx. 18 mo follow-up	On demand; Comprehensive Care: regular visits of 45–60 min. and phone/email contact; AIP: 1 motivational interviewing; school nurse agreement on medication administration (80%); pharmacy calls (76%), and PEF Device received (76%)	Family-oriented; in-person; in-clinic; phone calls; home spirometry; school nurse contact; pharmacist contact	Comprehensive care only (no AIP components)	Total days of healthcare outside the home*; School absence; Pulmonary function changes
Ng et al. (2021), Hong Kong/China [52]	Total n = 112; IG n = 56; CG n = 56	5 (4–11)	27%	Community hospital	Out-of- hospital support with digital access from community homes and community environments; in-hospital sessions	Web-based Home Asthma Education Program (WB-HAEP): online sessions with videos, games, and discussion; access via QR-code access; follow-up at the hospital	45 min. session with follow-up at 2 and 6 mo	Single-session of 45 min with ongoing unlimited access to web resources; usage (video/game viewing counts) tracked, 1 session/discussion with an asthma nurse	Family-oriented; in-person	Usual care	Parent knowledge, attitude, practice*; Parent anxiety; Asthma symptoms; Healthcare use; Asthma severity; Episode duration
Seid et al. (2010), USA [53]	Total n = 252; IG1 n = 81; IG2 n = 84; CG n = 87	7 (2–14)	39%	Community homes	Cross- sectoral community support	IG1: care coordination and education; IG2: care coordination plus problem-solving skill training after the care-coordination component	5–6 weeks	IG1: 5 sessions of 45–60 min/wk; IG2: 6 sessions of 45–60 min/wk	Family-oriented; in-person; in-home	Waitlist	Pediatric HRQOL*; Asthma-specific HRQOL; Symptom frequency; Healthcare utilization
Shames et al. (2004), USA [54]	Total n = 119; IG n = 59; CG n = 60	8 (5–12)	42%	NI	Out-of- hospital support	Multicomponent program: asthma management and care coordination; video game education; accompanied clinic appointments and hotline access	12 mo with 2, 8 and 12 mo follow-up	3 education sessions with case manager; 2 allergist visits; video game equipment; 18 h hotline access; home diary monitoring	Individual and self-guided; in-person; educational sessions in-clinic; phone support; nurse-led hotline; home-based video game learning	Usual care plus a nonviolent video game without asthma content	Asthma symptom days*; Lung function; Peak expiratory flow; Bronchodilator reversibility; Physical health; Child social activity; Family social activity; Emotional health; Child asthma knowledge; Parent asthma knowledge; Urgent care use
Wu et al. (2024), China [55]	Total n = 81; IG n = 40; CG n = 41	9 (6–11)	38%	Tertiary hospital outpatient clinic and community homes	Access embedded in everyday community context-adopted online platform	WeChat Mini Program: digital monitoring, education, and nurse feedback	6 mo	Daily logs; weekly professional review; monthly assessments; education 2 times/wk; daily communication window	Family-oriented; telehealth; monitoring with nurse-led supervision	Usual care	Childhood Asthma Control Test (c-ACT)*; Asthma control level; Number of exacerbations; Lung function
Valery et al. (2010), Australia [56]	Total n = 88; IG n = 35; CG n = 53	7 (1–17)	31%	Community clinics	Involvement of indigenous health workers and community environments	Indigenous asthma education add-on to usual care: education and support materials	6 mo	3 sessions at 1, 3, and 6 mo after baseline	Family-oriented; in-person; trained CHW-led; culturally tailored	Usual care	Number of unscheduled visits to hospital or doctor for asthma exacerbation*; Functional Severity Index; Asthma QoL; Carer medication knowledge and action plan interpretation; School days missed due to wheezing
Whitman et al. (1985), USA [57]	Total n = 38; IG n = 19; CG n = 19	9 (6–14)	35%	Community health education settings	Out-of- hospital support	Self-Care Rehabilitation (SCRPA): breathing, relaxation, conditioning, education, and coping training	1 mo	8 classes of 90 min.	Family and peer-oriented; in-person; in-group; SCRPA instructor-led classes for children and parents	Waitlist (delayed delivery)	Asthma episodes*; Asthma knowledge; Health locus of control; Attitude; Skills; Missed activities; School absence; Severity; Emergency visits
Bruzzese et al. (2006), USA [58]	Total n = 591; IG n = 307; CG n = 284	8 (5–11)	41%	Community public schools	Community neighborhoods and school staff involvement in care	School health team: multidisciplinary team with case detection, education, and coordination	24 mo	Monthly support for 12 mo; support on an as-needed basis for additional 12 mo	Family and peer-oriented; in-person; in-group; school nurse and school physician-led support; staff education via workshops; caregiver education	Usual care	Number of days with asthma symptoms*; Number of nights woken due to asthma; Days with activity limitation; Urgent clinician visits; ED visits; Hospitalizations; School absence; Caregiver QoL; Medication use; School attendance; Time on asthma tasks; Case detection yield
Bryant-Stephens et al. (2024), USA [59]	Total n = 626; IG1: n = 121; IG2: CHW in primary care n = 110; IG3: School IG with CHW n = 112 CG1: n = 112; CG2: n = 82; CG3: n = 89	9 (5–13)	42%	Public schools, community clinics and community homes	Cross- sectoral bridging multiple community settings by CHW	IG1, Open Airways for Schools+ (OAS+): CHW in primary care and at school (combined): education, home visits, and school-based management; IG2, CHW in primary care; IG3, CHW at school	12 mo	4 CHW home visits plus ongoing school and clinic components (school-based group education and therapy support and care coordination)	Caregiver-oriented; in-person; in-group; CHW-led delivery across clinical, home, and school settings	CG1: other (school) intervention; CG2: no intervention; CG3: usual care	Asthma Control Questionnaire*; Daytime symptoms; Nighttime symptoms; School absences; ED visits; Hospitalizations; Oral steroid use; Caregiver QoL
Cicutto et al. (2005), Canada [60]	Total n = 256; IG n = 132; CG n = 124	9 (6–11)	41%	Community public schools	School- centered community partnerships involving schoolteachers and parents	Roaring Adventures of Puff (RAP) school program: asthma self-management games and role-play	6 wks	6 sessions of 1 h/wk	Family-oriented, caregiver and peer-oriented; in-person; in-group; certified asthma educators; parental involvement in final session; homework	Usual care (delayed delivery)	Asthma-related QoL* and acute asthma healthcare use*; Self-efficacy; School absenteeism; Interrupted activity days; Parental work loss
Cicutto et al. (2013), Canada [61]	Total schools n = 170 (n = 1316 children); IG schools n = 85; CG schools n = 85	8 (7–10)	43%	Community public schools	Community asthma support at schools involving schoolteachers, nurses, and parents	Roaring Adventures of Puff (RAP) and school environment: student education plus school-wide asthma-friendly initiatives for school-wide policy and environmental changes	14 mo	6 sessions of 1 h/wk delivered over lunch hours; 8 school-level implementation activities over 14 mo	Family, caregiver and peer-oriented; in-person sessions; public health nurse-led with co-delivery by certified asthma educators	Waitlist (school-level)	Urgent asthma-related healthcare use*; School absenteeism; Activity limitation; Inhaler technique; Asthma-related QoL
Clark et al. (2004), USA [62]	Total n = 835; IG n = 416; CG n = 419	NI (grades 2–5; appx. 7–10 y)	NI	Community public schools in low-income communities	Community asthma support at schools involving schoolteachers, nurses, and parents	Open Airways for Schools (OAS): self-management education, enhancing peer-awareness and school training	6 wks with 24 mo follow-up	Multiple components across entire school	Family, caregiver and peer-oriented; classroom sessions; staff training; family events; take-home materials	Waitlist (delayed delivery)	Asthma symptoms*; Parent asthma management index; Academic grades; School-record absences; Parent-reported asthma-related absences
Clark et al. (1986), USA [63]	Total n = 310; IG n = 207; CG n = 103	9 (4–17)	36%	Community hospital	Out-of- hospital support in community homes	6-topic education program: parent–child sessions on disease management, prevention, and communication	6 wks	6 sessions/wk: 5 parent sessions, 5 child sessions and 1 joint family session	Family and peer-oriented; in-person; trained health educators-led	Usual care (medical routine)	Parent asthma management index *; Children’s self-reported management behaviors; children’s feelings (worry, shame) related to asthma and school
Clark et al. (2010), USA [64]	Total n = 1292; IG1 n = 468; IG 2 n = 416; CG n = 408	12 (10–13)	48%	Community public schools	Community asthma support at schools involving schoolteachers, and parents	IG1: Open Airways for Schools (OAS): self-management education IG2: OAS added a peer-awareness component	6 wks with 12 and 24 mo follow-up	IG1: 6 OAS sessions; IG2: 6 OAS sessions and 3 peer-led sessions and assemblies	Peer-oriented; in-person; in-group; delivered during regular class time	No intervention	Asthma symptoms (day/night)*; Pediatric Asthma QoL*; Self-regulation*; Academic grades*; Parent asthma management practices; Child asthma management practices
Eakin et al. (2020), USA [65]	Total n = 398; IG (ABC plus Head Start) n = 199; CG (Head Start only) n = 199	4 (2–6)	38%	Low- income communities	Community asthma education centers and community homes	School-based education and staff training (Head Start program) added home visits and booster phone calls	12 mo with booster phone calls at 3, 6, and 9 mo	4 home visits and 3 booster calls	Family and peer-oriented; in-home education; booster phone calls; school-based education	Other treatment (school-based education alone)	Asthma control (TRACK questionnaire)*; Oral corticosteroid courses; ED visits; Hospitalizations; Caregiver QoL; Environmental assessment; Healthcare use
Everhart et al. (2024), USA [66]	Total n = 250; full IG n = 118; partial IG n = 69; CG n = 63	7 (5–11)	41%	Community public schools and community homes	Cross- sectoral community settings involving CHW, schools and caregivers	RVA Breathes program: CHW, home remediation and school-based education, environmental changes, and care coordination	9 mo with 3, 6, and 9 mo follow-up	4 multicomponent sessions delivered every 2–3 mo by CHW	Family-oriented; in-home sessions; coordination letters and documentation to schools and providers; culturally tailored materials	Usual care enhanced (added publicly available asthma information materials)	Healthcare utilization*; Childhood Asthma Control Test; Days with asthma symptoms; Child asthma QoL; Caregiver QoL; Prescribed controller medication; Asthma action plan presence
Glasgow et al. (2003), Australia [67]	Total n = 174; IG n = 101; CG n = 73	NI (5–12)	45%	GP	Cross- sectoral community settings involving CHW, GP and caregivers	3+ plan: structured asthma care follow-up with GP; patient education and written action plans	12 mo with 6 and 12 mo follow-up	“3+ plan” targeted delivery over 3 or more consultations; family reminders to attend	Individual and family-oriented; GP-led	Usual care (GP)	GP consultations*; Action plan; ED attendances; Symptom-free day score; Lung function; School absence; Wheeze episodes; Activity limitation; Medication use; Device use
Horner et al. (2014), USA [68]	Total n = 183; IG n = 101; CG n = 82	9 (NI, grades 2–5: appx. 7–12 y)	NI; balanced across groups	Rural communities	Community public schools and community homes	The Asthma Health Education Model: short child sessions and home-based parent education	5,5 wks with 1 mo follow-up	16 short lunchtime sessions for children; 1 home visit for parents	Individual and family-oriented; school nurse-led child sessions; research staff-led home visits for parents; culturally tailored materials	Waitlist (delayed delivery)	Asthma episodes*; Asthma knowledge; Health locus of control; Parental attitudes; Skills performance; Missed activities; School absence; Emergency visits
Kintner et al. (2009), USA [69]	Total n = 66; IG n = 38; CG n = 28)	11 (9–12)	48%	Community public schools	Community school-based asthma management with academic support	SHARP: Student school-based education and community-component (counseling) for caregivers	10 wks	10 student sessions of 50 min (500 min total) plus 1 caregiver session of 3 h; written materials (workbook); community event; phone access (optional extra contacts not specified)	Family and caregiver-oriented; in-person; in-group; instruction and counseling led by trained staff	Usual care	Asthma knowledge*; Asthma management*; Asthma acceptance*; Vigilance*; Activity participation
Kintner et al. (2015), USA [70]	Total n = 205; IG n = 117; CG n = 88	10 (9–12)	40%	Community public schools in low-income community	School- settings involving school staff and peers	SHARP program (replication): structured educational and counseling activities; workbook-guided sessions supporting asthma acceptance	10 wks with 1, 12, and 24 mo follow-up	10 sessions of 50 min.	Family and caregiver-oriented; in-person; in-group; teacher-led counseling embedded into academic learning	Other intervention (Open Airways for Schools: 6 sessions of 50 min., nonacademic asthma education and handouts)	Acceptance of asthma questionnaire*; Openness to learning; Openness to sharing; Vigilance; Taking control; Connectedness with friends; Connectedness with classmates; Connectedness with classroom teachers; Connectedness with PE teachers
Levy et al. (2006), USA [71]	Total n = 243; IG n = 115; CG n = 128	NI (6–10)	43%	Community public schools	Community teams including school nurses, administrators, and families to reduce absences and hospitalizations	Open Airways, Nurse Case Management: education groups, monitoring, and coordination	1 school year (appx.8 mo)	Weekly sessions; nurse present 1 day/wk per school	Individual and family and caregiver-oriented; in-person; in-group; school-based sessions with phone follow-up	Usual care (standard school nurse services)	School absences*; Hospitalizations*; Nighttime symptoms; Asthma knowledge
McQuaid et al. (2025), USA [72]	Total n = 433; Cluster 1: poor asthma control n = 356; Cluster 2: not well controlled n = 77 (own control)	7 (2–12)	44%	Community public schools and community homes	City-wide community strategy involving caregivers, CHW, and school partners	Controlling Asthma in Schools Effectively (CASE) plus Home Asthma Response Program (HARP) Model: school education, home visits, remediation, and coordinated action plans.	Appx. 1 year (variable depending on assignment); CASE during school year; HARP 3 sessions; 3, 6, 9, and 12 mo follow-up	CASE: In-school education; after-school caregiver sessions; staff training; environmental assessment; HARP: 3 CHW home visits	Individual and family and caregiver-oriented; in-person; in-group; school-based (child, caregiver, staff); in-home CHW sessions; shared information system for care coordination	Each cluster served as its own control	Asthma control at 3 mo*; c-ACT; Respiratory and Asthma Control score (TRACK)
MeGhan et al. (2003), Canada [73]	Total n = 162; IG n = 76; CG n = 86	NI (5–13)	41%	Community public schools	School- settings involving school staff and peers	Roaring Adventures of Puff (RAP): interactive self-management training using behavioral theory	Appx. 6 mo (1 school term) with 9 mo follow-up	6 child sessions of 60 min.; parent/teacher awareness events (frequency not quantified)	Family, peer and caregiver oriented; in-person; in-group; interactive child instruction; parent/teacher events; instructor–physician communication letters; school environmental recommendation	Usual care (medical only)	Asthma management*; Health status; QoL*; Asthma severity; Asthma symptoms; Medication use; Healthcare utilization; Absenteeism; Self-efficacy; Unscheduled doctor visits; Emergency visits; Missed school days; Limitations in play; Preventer medication use; Reliever medication use; Peak flow meter use; Asthma action plan possession; Parental understanding; Parental control; Parental coping
Tieffenberg et al. (2000), Argentina [74]	Total n = 188; IG = 127; CG = 61 asthma (excluding n = 167 participants diagnosed with epilepsy	NI (6–15)	NI	Community public schools and community centers	Cross- sectoral community settings involving community non-clinical spaces, and families	ACINDES program: autonomy and communication for families; play-based educational activities for children; integrating health and educational environments; families and teachers actively engaged	5 wks with 6 and 12 mo follow-up	5 sessions (1 session/wk) of 2 h plus 1 booster-session at 2–6 mo (12 h in total)	Individual and family-oriented; in-group (children and children–parents groups)	Usual care	Children’s Health Locus of CG*; Adult Health Locus of Control*; Sociocultural factors; Crises; Routine visits*; Emergency visits*; Hospitalizations; School absenteeism
Bruzzese et al. (2008), USA [75]	Total n = 24; IG n = 12; CG n = 12	13 (NI; grades 6–8: appx. 11–14 y)	46%	Community public schools	Community asthma management involving schoolteachers and families	Asthma: It’s a Family Affair! program: parallel sessions on self-management and parenting strategies for students and caregivers	6 wks for students; 5 wks for caregivers	1 session/wk	Family and caregiver-oriented; in-person; in- group; clinical psychologist-led	Waitlist (delayed delivery)	Asthma management behaviors*; Responsibility; Symptom severity; Family relations
Celano et al. (2012), USA [76]	Total n = 43 (analyzed n = 41); IG n = 21; CG n = 20)	11 (8–13)	37%	Urban low-income communities	Community homes	Home-Based Family Intervention (HBFI): tailored asthma and psychosocial modules with technique feedback	4 mo	4–6 home visits of 64–96 min.	Family-oriented; in-person; two-person team (a psychology postdoctoral fellow and respiratory therapist within “Asthma Counselors” program)	Usual care enhanced (1 home visit by respiratory therapist; asthma action plan; devise use monitoring; home-trigger control)	FAMSS*; Inhaler technique; Asthma morbidity; Tobacco smoke exposure; Caregiver stress
Everhart et al. (2017), USA [77]	Total randomized n = 61 (analyzed n = 28); IG n = 15; CG n = 13	10 (7–12)	32%	Urban community or research office	Access to community homes and community environments through a mobile platform	Ecological momentary assessment (EMA) feedback: mobile monitoring with personalized stress feedback and problem-solving	2 wks plus 1 feedback session with 4 wks and 4 mo follow-up	14 days with EMA (twice daily) and prompts over 14 days; 1 feedback session of 60 min.	Family-oriented; hybrid: digital (smartphone) EMA data collection and in-person feedback	Other intervention (general child health information and two lifestyle goals for general well-being)	Caregiver emotional health (QoL, stress, positive affect, sleep, mood, social support, neighborhood safety)*; Asthma Control: cACT (ages 7–11) or ACT (age 12)
La Roche et al. (2006), USA [78]	Total allocated n = 35 (randomized n = 24; IG1 n = 12; IG2 n = 12); added CG group n = 11 (non-randomized; matched on age, sex, ethnicity)	10 (7–13)	41%	Community health center	Community health service for minority populations	IG1, Multi-Family Asthma Group Treatment (MFAGT): culturally tailored group intervention; IG2: Standard Psychoeducational Asthma Intervention (SPAI): standard psychoeducation through structured didactic teaching	3 sessions with 12 mo follow-up	3 sessions of 1 h each delivered on separate days	Family-oriented; in-person; in-group; interactive delivery; culturally adapted (IG1)	No intervention	Number of asthma-related ED visits*; Individualism–Collectivism; Asthma knowledge; Asthma skills
McGhan et al. (2010), Canada [79]	Total n = 287 (analyzed n = 266; IG n = 104; CG n = 162	9 (6–13)	38%	Community public schools	School-based asthma management involving teachers and parents	Roaring Adventures of Puff (RAP): no structured asthma education on self-management and communication; parent–teacher events	6 wks	6 sessions (1 session/wk of 45–60 min.) plus one optional 2 h parent/teacher evening	Family, peer and caregiver-oriented; in-person, in-group; trained respiratory therapist and a community health nurse-led	Usual care	Pediatric Asthma QoL Questionnaire (PAQLQ)*; Asthma management behaviors; Healthcare use; Activity limitation
**T1D**											
Mitchell et al. (2018), UK [80]	Total n = 20; IG n = 10; CG n = 10	12 (7–16)	60%	Community clinics and community homes	Tailored approach including local sports and activity opportunities and community environments	ActivPals program: physical activity intervention with goal-setting, monitoring, and digital tools	4 wks	Single initial PA consultation; daily self-monitoring; ongoing text prompts; pedometer app syncing	Individual and family-oriented; in-person PA consultation; remote support via text messaging and digital self-monitoring	Waitlist (delayed delivery)	Physical activity and sedentary behavior*; QoL; Anthropometrics
Patton et al. (2024), USA [81]	Total n = 34; IG1 n = 13; IG2 n = 11; IG3 n = 10 (Between-group comparison)	10 (8–12)	47%	Community clinics and community homes	Designed through user involvement (parental/ family everyday challenges) for contents	Remedy to Diabetes Distress (R2D2): telehealth CBT-based modules for diabetes distress with tracking and coping tools; motivational interviewing; tailored to family routines; parent–child dyads engaged	10 wks	3 intensities: IG1 (self-guided): access to materials only; IG2: access to materials and 3 telehealth sessions (30 min. each); IG3: access to materials and 8 telehealth sessions (30–45 min. each)	Individual and family-oriented; hybrid digital and in-person/in-group: home-based materials, in-person motivational interviewing, and supervised group sessions; delivered by volunteer healthcare students	Active comparators	Diabetes Distress in Parents and Children*; HbA1c; Treatment satisfaction; Feasibility outcomes
Quirk et al. (2018), UK [82]	Total n = 12; IG n = 8; CG n = 4	10 (9–11)	54%	Community clinic and community homes	Anchored in community sports and activity environments	Steps to Active Kids with Diabetes (STAK-D): multicomponent PA program with pedometer tracking, diaries, and motivational interviewing	6 wks	1 session/wk; engagement varied across components	Individual, self-guided and family and peer-oriented; in-person; in-group; individual motivational interview at home	Usual care (limited structured PA support)	Physical activity*; Self-reported physical activity; Self-efficacy; Acceptability; Feasibility Outcomes
Bloomfield et al. (1990), UK [83]	Total n = 48; IG n = 24; CG n = 24	9 (6–12)	52%	Community health center adjacent to hospital	Out-of-hospital care with active parental and local educators’ involvement	Diabetic club: group education with activities, discussions, and monitoring	12 mo with 12 mo follow-up	10 sessions of 3.5 h (avg. 22 h)/year	Individual and self-guided; in-person; in-group (separate for parents and children); practical activities	Usual care	Diabetic Control*; Hospital admissions; Hypoglycemic episodes; Insulin dose; School absence; Blood glucose testing; Injection site rotation; Dietary intake; Diabetes factual knowledge; Diabetes problem-solving ability; Child diabetes knowledge; Psychological functioning; Parental perceptions
Chernoff et al. (2002), USA [84]	Total n = 161 randomized (analyzed n = 136); IG n = 72; CG n = 64	9 (7–11)	NI	Community clinic	Community family support involving parent mentors	Family-to-Family Network: Child Life Specialists (CLS) together with experienced mothers; home visits, peer support, and coordinated family education; support letters for children	15 mo	Max. 7 CLS visits of 60–90 min. during the program; coordination of CLS-parent mentor visits; avg. 3 (0–13) visits from educated Child Life Specialist; avg. 2 (range 0–6) visits from parent mentor; 1 (0–6) special event; avg. 3 (0–11) significant educated Child Life Specialists’ calls; avg. 7 (0–43) significant calls from parent mentor; avg. 15 (5–28) support letters received	Individual and family-oriented; in-person; home visits; phone calls; mailed letters; in-person family events; professional-parent (mothers) team delivery	Other intervention (received name and phone number of parent mentor for optional support)	Child’s Psychosocial Adjustment*; Depression; Anxiety; Self-esteem
Ellis et al. (2007), USA [85]	Total n = 127; IG n = 64; CG n = 63	13 (10–17)	51%	University clinic and community schools and homes	Cross- sectoral out-of-hospital care	Multisystemic therapy (MST): intensive family therapy addressing multilevel diabetes management	6 mo	Therapists met families ≥2–3 times/wk	Individual and family-oriented; in-person; in home-, school, and community, settings	Usual care (quarterly routine multidisciplinary visits)	Family Relationship Index (Caregiver support)*; Diabetes-specific caregiver support; Adherence; Metabolic control
Ellis et al. (2012), USA [86]	Total n = 146; IG n = 74; CG n = 72	14 (10–18)	56%	University clinic	Cross- sectoral out-of-hospital care	Multisystemic therapy: home-based family therapy targeting adherence and system barriers; therapist attendance at medical visits; skills practice contacts; meetings with school/clinic staff	6 mo and 6 mo follow-up	Mean 45.7 sessions over 5.6 mo; min. ≥2 contacts/wk; session length variable (brief skills practices—15 min.; school staff trainings—1–2 h; clinic visits ≥2 h); treatment ended when goals met rather than fixed session count	Individual and family-oriented; in-person; in-home, community, and school settings	Other intervention (30 min. of phone support/wk)	Metabolic control*; Adherence; Adverse events
Flores Garcia et al. (2024), USA [87]	Total randomized n = 82; (analyzed n = 79); IG1 n = 16; CG1 n = 23; CG2 n = 19; CG3 n = 21	13 (10–17)	47%	Community outpatient clinic	Designed by user involvement for marginalized communities	Team Clinic (virtual groups and patient-centered care): telehealth support/education; separate youth and family sessions using motivational interviewing	15 mo with 12 mo follow-up assessments	4 sessions of 1 h every 3–5 mo	Family and peer-oriented; in-group telehealth	Usual care or other intervention: CG1, usual care plus virtual group; CG2, patient-centered care (routine clinic appointments with shared decision-making), no virtual component; CG3, usual care	Depressive symptoms*; Diabetes distress; Diabetes strengths and resilience; Family conflict; Family responsibility; Healthcare climate; HbA1c; Device use; Attendance; Satisfaction
Graue et al. (2005), Norway [88]	Total n = 101; IG n = 55; CG n = 46	14 (11–17)	47%	Community outpatient clinic	Community-like support environment promoting peer-contact	Group sessions plus individualized nurse-led consultations; age-stratified groups; parallel parent groups; routine access to dietician; psychologist; social worker in clinic	15 mo	Group visits at 0; 6; 12 mo (3 × 3 h; 4–9 participants per group); individual consultations at 3; 9; 15 mo (3 × 45 min)	Family and peer-oriented; in-person; in-group education and counseling; individual nurse-led computer-assisted consultations	Usual care	Metabolic control*; Diabetes-specific QoL*; Generic health-related QoL*; Satisfaction; Hypoglycemia; DKA episodes; Hospital admissions; Anthropometrics; Treatment regimen
Gregory et al. (2019), UK [89]	Total n = 203; IG n = 101; CG n = 102)	10 (8–17)	46%	Community diabetes centers	Home-based care with active involvement of family and local environment	Home-based education and insulin initiation with monitoring	24 mo	Home arm: no overnight admission	Family-oriented; in-group; in-home multidisciplinary education and support	Usual care (hospital admission with similar content delivered on-ward and outpatient follow-up)	HbA1c*; Child QoL; Self-esteem; Parent outcomes; Growth outcomes; Resource use; Work/school absence
Grey et al. (2013), USA [90]	Total n = 320; IG n = 167; CG n = 153	12 (11–14)	55%	University diabetes centers	Web-based program accessible from home or community spaces	TeenCope and Managing Diabetes: online coping skills and diabetes education programs	5 wks with 3, 6, and 12 mo follow-up	5 sessions of 30 min.	Individual; hybrid: telehealth/in-person; web-based modules plus monitored discussion board (TeenCope).	Other intervention (web-based education/problem solving)	HbA1c and QoL*; Perceived stress; Stress responses; Self-efficacy; Self-management; Social acceptance; Family conflict; Family responsibility; Healthcare climate; Attendance; Satisfaction
Hanberger et al. (2013), Sweden [91]	Total n = 474; IG n = 244; CG n = 230	13 (0–18)	51%	Community diabetes clinics	Web-based program accessible from home or community spaces	Diabit portal: web platform for communication, peer interaction, and education	12 mo with 12 mo follow-up	Self-directed use; mean 4.4 site visits (year 1) and 4.7 (year 2); active users defined retrospectively as ≥5 logins in first access year	Individual, self-guided and peer-oriented; telehealth; asynchronous information and peer networking; occasional newsletters/links to new content; no structured education sessions tied to portal use	Usual care	HbA1c and QoL*; HRQOL*; Empowerment; Quality of care; Severe hypoglycemia; Blood glucose monitoring frequency
Hilliard et al. (2022), USA [92]	Total families n = 157; IG n = 115; CG n = 42	5 (1–6)	55%	Community diabetes centers	Designed for home use and follow-up within family and community	Stepped-care behavioral intervention designed to support parents’ psychosocial functioning and promote children’s glycemic outcomes; trained parent coach involved	9 mo with 15 mo follow-up	3 intervention steps, each increasing in intensity: (1) peer support from parent coach during 9 mo; (2) 5 phone sessions (4 individual; 1 in-group) within 3 mo; (3) 2 consultations with diabetes educator/psychologist)	Individual and peer-oriented; hybrid: telehealth/in-person; remote (phone/text/email) peer coaching; phone-based counseling; in-person or telehealth for educator/psychologist consults	Usual care	Parent depressive symptoms*; Child HbA1c*; Satisfaction; Fidelity; Session completion; Adherence
Katz et al. (2014), USA [93]	Total n = 153; IG1 n = 51; IG2 n = 52; IG3 n = 50 (between-group comparison)	13 (8–17)	56%	Community diabetes clinic	Community in-clinic support to diabetes care through non-medical involvement	Care Ambassador model: care coordination with enhanced communication and psychoeducation	25 mo	3 intensities: IG1: usual care and basic care coordination; IG2: as IG1 added monthly outreach by phone or mail; IG3: as IG2 added 4 quarterly sessions of 30 min. with psychoeducation; all arms had avg. 9–10 visits/2 years	Family-oriented; in-person, in-clinic; delivered by trained non-medical Care Ambassador	Active comparators	Glycemic control*; Parental involvement; Family conflict; QoL; Blood glucose monitoring frequency
Majidi et al. (2021), USA [94]	Total n = 91 (analyzed n = 86); IG n = 44; CG n = 42	12 (11–13)	51%	Large free-standing academic diabetes centers	Community in-clinic peer-support system	Team Clinic: shared appointments (group visits) with education, structured goal-setting, motivational interviewing and shared decision-making; patient-centered care approach for families	12 mo	4 quarterly visits of 45–60 min.; additional brief individual time at each visit; separate caregiver group per visit; topics rotated each quarter	Individual and family and peer-oriented; in-person; in-group; in-clinic; multidisciplinary facilitators	Usual care (similar without group sessions)	Depression*, emotional problems*, family conflict* (DFCS); Caregiver depressive symptoms; HbA1c; Time in range; Attendance; device (glucose monitor) adoption
Nansel et al. (2007), USA [95]; Nansel et al. (2009), USA [96]	Total n = 81; IG n = 40; CG n = 41 [24 mo follow-up: Total n = 78/81; IG n = 39/40; CG n = 39/41]	14 (11–16)	56%	Community diabetes clinic	Home and community-based care with community involvement	Behavioral sessions with motivational interviewing, goal-setting, and parental involvement	2 mo with 6 and 12 mo follow-up	6 sessions with supplemental on-phone contact	Individual and family-oriented; in-person; one-on-one sessions with youth; initial joint session with parent; delivered trained nonprofessionals at home or public locations; phone follow-ups	Other intervention (educational materials)	HbA1c*; Adherence; Self-efficacy; Outcome expectations; QoL Follow-up: A1C*
Sundelin et al. (1996), Sweden [97]	Total n = 38; IG n = 19; CG n = 19	8 (3–15)	63%	Community hospital	Homelike training apartments	Intensive family crisis program with family-system therapy and transition support post-discharge; environmental and social-ecological support; medical and psychosocial care integrated	2 wks with 1 wk, 1 mo, 6 mo, 12 mo, and 24 mo follow-up	High-intensity early intervention: daily living in treatment apartment with multiple professional visits	Family-oriented; in-person; in-group; delivered by doctors, nurses, social worker and psychologist	Usual care	Cognitive/Psychosocial development *; HbA1c; Time spent with staff; Family structure changes
Christie et al. (2016), UK [98]	Total n = 327; IG n = 159; CG n = 168	13 (8–16)	55%	Community diabetes clinics	Out-of- hospital diabetes care	CASCADE: group diabetes education using motivational interviewing and solution-focused therapy	4 mo	4 sessions of 2 h each for groups of 3–4 families	Family and peer-oriented; in-person; in-group; delivered in clinical environments with community access	Usual care	HbA1c*; QoL; Psychosocial impact; Diabetes responsibility; Hypoglycemic episodes; Hospital admissions; Health service utilization; Clinic attendance
Ellis et al. (2005), USA [99]; Ellis et al. (2008), USA [100]	Total n = 127; IG n = 64; CG n = 63	13 (10–17)	41%	Community diabetes clinics	Cross- sectoral outreach involving local systems around adolescent	Multisystemic therapy for diabetes: family-centered CBT-based intervention across multiple systems targeting adherence and care coordination	6 mo with 24 mo follow-up	2–3 sessions/wk	Family-oriented; in-person; in-group; therapist-led; delivered at-home and in the community; therapist-accompanied medical visits	Usual care	HbA1C*; Blood glucose testing frequency; Insulin adherence; Dietary adherence; Hospital utilization; Emergency department visits; Inpatient admissions Follow-up: DKA hospital admissions*; Costs
Ellis et al. (2019), USA [101]	Total n = 50; IG n = 26; CG n = 24)	14 (10–18)	62%	Tertiary health center and community homes	Cross- sectoral involvement (CHW and local organizations)	REACH for Control: CHW- delivered tailored modules with care coordination and follow-up	6 mo	2 sessions/wk; 20 primary and 16 brief follow-up sessions	Individual and family-oriented; in-person; in-group; at-home and in-the-community delivery by trained CHW	Usual care	Glycemic control*; Regimen adherence; Blood glucose testing frequency; QoL; Treatment dose; Satisfaction
Jewell et al. (2025), USA [102]	Total n = 16; IG n = 8; CG n = 8)	9 (2.3–12.8)	63%	Community diabetes clinic	Rural communities	Occupation-based coaching: structured coaching conversation; family-centered problem-solving for daily routines	3 mo	1 session/wk of 60 min.; continuous glucose monitoring where applicable	Family-oriented; hybrid: telehealth/in-person (video visits)	Usual care (delayed delivery)	Parenting Sense of Competence*, WHOQOL-BREF *, Goal Attainment Scale*, Caregiver engagement/Caregiver talk*, Evidence of Independent Capacity Rating Scale (EICRS)*; HbA1c
**JIA**											
Stinson et al. (2010), Canada [103]	Total n = 46; IG n = 22; CG n = 24	15 (12–18)	67%	Community rheumatology clinic	Out-of- hospital care with user-informed contents	Teens Taking Charge: disease-management education with coaching calls	12 wks	1 session/wk of 20–30 min. added weekly structured phone coaching of avg. 17 min.; optional parent modules	Individual and self-guided; in-home; telehealth with self-paced modules; on-phone contact	Other intervention (weekly brief phone calls)	Juvenile Arthritis QoL Questionnaire*; Pain intensity; Perceived stress severity; Adherence; Self-efficacy; JIA-specific knowledge
Stinson et al. (2020), Canada [104]	Total randomized n = 333 (analyzed n = 219; IG n = 169; CG n = 164	14 (12–18)	70%	Community hospital	Out-of- hospital care	Teens Taking Charge expanded: web modules, coaching, and caregiver education for disease-management	3 mo with 6 and 12 mo follow-up	1 module/wk (12 modules total); automated weekly reminder emails; monthly coach calls to adolescent	Individual and family-oriented; in-home; telehealth with self-paced modules; on-phone contact	Other intervention (website access, on-phone reminders and standard coaching)	Pain intensity*; Pain interference*; Health-related QoL*; Emotional symptoms; Adherence; Coping; Knowledge; Self-efficacy

* Ref. primary outcome(s). Abbreviations: Appx., approximately; CG, control group; CHW, community health worker; GP, general practice; IG, intervention group; JIA, juvenile idiopathic arthritis; mo, month/months; NI, no information; PA, physical activity; PEF, peak expiratory flow; T1D, type 1 diabetes; QoL, quality of life; wk, week.

**Table 3:** Intervention deliverers.

Background/Profession	N (%) Represented in n = 71 Interventions *	As Primary Deliverers	Involved in the Delivery
**Heath professionals**			
Nurses	25 (35)	18 (25)	7 (10)
Trained therapists, unspecified	10 (14)	10 (14)	-
Physicians	8 (11)	5 (7)	3 (4)
General practitioners	5 (7)	3 (4)	2 (3)
Psychologists/psychology students	7 (10)	5 (7)	2 (3)
Dietitians	5 (7)	1 (1)	4 (6)
Occupational therapists	2 (3)	1 (1)	1 (1)
Physiotherapists	1 (1)	-	1 (1)
Pharmacists	1 (1)	1 (1)	-
**Other, non-medical professionals**			
Staff trained within a specific intervention	20 (28)	18 (25)	2 (3)
Researchers	6 (8)	6 (8)	-
Schoolteachers	3 (4)	3 (4)	-
Social workers	2 (3)	1 (1)	1 (1)
Orthotic technicians	1 (1)	-	1 (1)
NI	2 (3)	2 (3)	-
**Lay persons**			
CHW	14 (20)	13 (18)	1 (1)
Peer parents	4 (6)	3 (4)	1 (1)
Young peers	1 (1)	1 (1)	-

Note. CHW, community health worker; N, number; NI, no information. * The counts are non-mutually exclusive. As several of the sample interventions were delivered by multidisciplinary teams, the total number of representations is higher than the total n = 71 included interventions.

## Data Availability

The original contributions presented in this study are included in the article. Further inquiries can be directed to the corresponding author.

## Supplementary Materials

The following supporting information can be downloaded at https://www.mdpi.com/article/10.3390/children13091232/s1. Search Blocks and Terms and Search Strategy.
