# Supplementary material for: Mapping Randomized Controlled Trials of Family- and Community-Centered Healthcare Interventions for Pediatric Asthma, Type 1 Diabetes, or Juvenile Idiopathic Arthritis—A Scoping Review
Source: Children (Basel). 2026 Sep 11;13(9):1232. doi: 10.3390/children13091232 (PMC13605961; doi:10.3390/children13091232)
Supplement: Supplementary file 1 [file children-13-01232-s001.zip › children-4526927-supplementary.pdf]

Search Blocks & Terms

CHILD & ADOLESCENT

MESH terms/Headings:

Child/ or Child Health/ or Child Health Care/ or Child Health Services/ or Child Support/ or Adolescent/ or Adolescent Health/ or Adolescent Health Services

Keywords ab,kf,ti. (.kf=.tw In PsycINFO):

Child* OR child health OR Child Health Care OR child health services OR Rehabilitation pediatric OR pediatric rehabilitation OR Adolescen* OR Adolescent Health OR adolescent health services OR youth* OR teen*

FAMILY

MESH terms/Headings:

Family/ or Family Health/ or Family Care/ or Family centered care/ or Family Support/ or Family Nursing OR family intervention OR family members OR Parents OR Siblings

Keywords ab,kf,ti. (.kf=.tw In PsycINFO):

Family OR families OR family health OR family research OR family nursing* OR nursing*, family OR family-centered nursing* OR family centered nursing* OR nursing*, family-centered OR nursing*, family centered OR pediatric nursing* OR nursing*, pediatric OR children and young people's nursing OR school nursing* OR nursing*, school OR research family OR family support* OR support family OR dyad* OR family-based OR family-centered OR family-oriented OR family-anchored OR family functioning OR family coping OR family based OR family centered OR family oriented OR family anchored OR family centered care OR family interaction OR family intervention* OR family member* OR Parent* OR Sibling*

COMMUNITY

MESH terms/Headings:

Community Health Services/ OR Community Care/ or Community Involvement

Community Support/ or Social Support/

Community Health Nursing/

Community Networks/ or Social Networks/ or Social Networking

Community Participation/

Social Prescribing/

School Health Services/ or School Based Intervention

Community-Based Participatory Research/

Keywords ab,kf,ti. (.kf=.tw In PsycINFO):

Community health service* OR health service*, community OR service*, community health OR community health care OR health care, community OR community healthcare OR healthcare, community OR Social Support OR social care OR Communit* OR neighborhood* OR Community Support* OR Support Community OR Community Network* OR Community Care Network* OR Network*, Community Care OR Care Network*, Community OR network*, community care OR Community Health Network* OR Health Network*, Community OR Network*, Community Health OR Community Participation OR Participation, Community OR Involvement*, Community OR Community Involvement* OR Community Action* OR Action*, Community OR Social Prescribing OR Prescribing, Social OR School health OR school health service* OR health service*, school OR service*, school health OR school-based health service* OR health service*, school-based OR service*, school-based health OR school based health service* OR health service*, school based OR service*, school based health OR school health promotion* OR health promotion*, school OR promotion*, school health OR community-based participatory research OR community based participatory research OR participatory research, community-based OR participatory research, community based OR community care OR community care service OR community health action OR community health care OR participatory research OR social network* OR network*, social OR online social network* OR social network*, online OR intervention*, school based OR intervention*, school-based

ASTHMA (childhood asthma)

MESH terms/Headings:

Asthma/

Keywords ab,kf,ti. (.kf=.tw In PsycINFO):

Asthma* OR bronchial asthma OR asthma bronchial* OR asthma pulmonal* OR chronic asthma OR lung allerg* OR asthma, bronchial OR asthmatic OR bronchus asthma OR childhood asthma OR early onset asthma

DIABETES TYPE 1 (childhood diabetes)

MESH terms/Headings:

Diabetes Mellitus, Type 1

Keywords ab,kf,ti. (.kf=.tw In PsycINFO):

diabetes mellitus, type 1 OR type 1 diabetes OR insulin-dependent diabetes mellitus OR juvenile-onset diabetes mellitus OR insulin-dependent diabetes mellitus 1 OR juvenile-onset diabetes OR diabetes type 1 OR childhood diabetes OR diabetes, type 1 OR diabetes mellitus, insulin-dependent OR diabetes mellitus, insulin dependent diabetes mellitus, juvenile-onset OR diabetes mellitus, juvenile onset OR diabetes mellitus, type I OR diabetes mellitus, insulin-dependent, 1 OR insulin dependent diabetes mellitus 1 OR diabetes, juvenile-onset OR juvenile onset diabetes OR diabetes, autoimmune OR autoimmune diabetes OR diabetes mellitus, brittle OR type 1 diabetes mellitus OR Diabetes, Autoimmune OR Autoimmune Diabetes OR iddm OR Diabetes Mellitus, Insulin-Dependent OR Diabetes Mellitus, Insulin-Dependent, 1 OR Diabetes, Type 1 OR Insulin Dependent Diabetes Mellitus 1 OR insulin dependent diabetes mellitus OR juvenile onset diabetes mellitus OR early onset diabetes mellitus OR diabetes mellitus type 1 OR diabetes mellitus type 1

JUVENILE IDIOPATHIC ARTHRITIS

MESH terms/Headings:

Arthritis, Juvenile/ or Juvenile Rheumatoid Arthritis

Keywords ab,kf,ti. (.kf=.tw In PsycINFO):

Arthritis OR juvenile idiopathic arthritis OR juvenile arthritis OR arthritis, juvenile OR idiopathic arthritis, juvenile OR arthritis, juvenile idiopathic OR juvenile chronic arthritis OR chronic arthritis, juvenile OR arthritis, juvenile chronic OR juvenile rheumatoid arthritis OR arthritis, juvenile rheumatoid OR rheumatoid arthritis, juvenile OR childhood arthritis OR arthritis, childhood OR arthritides, childhood OR childhood arthritides OR systemic arthritis, juvenile OR arthritis, juvenile systemic OR juvenile systemic arthritis OR Rheumatoid Factor Positive Juvenile Polyarthritis OR Rheumatoid Factor Negative Juvenile Polyarthritis OR polyarthritis, juvenile, rheumatoid factor negative OR polyarthritis, juvenile, rheumatoid factor positive OR polyarticular juvenile idiopathic arthritis OR pcjia OR pjia polyarticular juvenile idiopathic arthritis OR polyarticular-course juvenile idiopathic arthritis OR JIA OR juvenile arthritis deformans OR arthritis deformans juvenilis OR juvenile arthropathy OR arthropathy, juvenile OR reactive arthritis OR Arthritis reactive

Search Strategy

MEDLINE

Ovid MEDLINE(R) ALL <1946 to September 15, 2025>	
1	Family Health/ or Family/ or Family Nursing/ or Family Support/
2	Family Health/
3	Family/
4	Family Support/ or Family/
5	Family/ or Family Nursing/ or Family Health/
6	Child/ or Child Care/ or Child Health Services/ or Child Health/
7	Child Health/ or Child/ or Child Health Services/
8	Adolescent Health Services/ or Adolescent/ or Adolescent Health/
9	Adolescent Health Services/ or Adolescent/ or Adolescent Health/
10	Adolescent Health Services/
11	(Child* or child health or Child Health Care or child health services or Rehabilitation pediatric or pediatric rehabilitation or Adolescen* or Adolescent Health or adolescent health services or youth* or teen*).ab,kf,ti.
12	6 or 7 or 8 or 9 or 10 or 11
13	Community Health Services/
14	Community Support/ or Social Support/
15	Social Support/
16	Community Health Nursing/
17	Community Networks/
18	Community Participation/
19	Social Prescribing/
20	School Health Services/
21	Community-Based Participatory Research/ or Community Participation/
22	Asthma/
23	(Asthma* or bronchial asthma or asthma bronchial* or asthma pulmonal* or chronic asthma or lung allerg* or asthma, bronchial or asthmatic or bronchus asthma or childhood asthma or early onset asthma).ab,kf,ti.
24	Diabetes Mellitus, Type 1/

25	(diabetes mellitus, type 1 or type 1 diabetes or insulin-dependent diabetes mellitus or juvenile-onset diabetes mellitus or insulin-dependent diabetes mellitus 1 or juvenile-onset diabetes or diabetes type 1 or childhood diabetes or diabetes, type 1 or diabetes mellitus, insulin-dependent or diabetes mellitus, insulin dependent diabetes mellitus, juvenile-onset or diabetes mellitus, juvenile onset or diabetes mellitus, type I or diabetes mellitus, insulin-dependent, 1 or insulin dependent diabetes mellitus 1 or diabetes, juvenile-onset or juvenile onset diabetes or diabetes, autoimmune or autoimmune diabetes or diabetes mellitus, brittle or type 1 diabetes mellitus or Diabetes, Autoimmune or Autoimmune Diabetes or iddm or Diabetes Mellitus, Insulin-Dependent or Diabetes Mellitus, Insulin-Dependent, 1 or Diabetes, Type 1 or Insulin Dependent Diabetes Mellitus 1 or insulin dependent diabetes mellitus or juvenile onset diabetes mellitus or early onset diabetes mellitus or diabetes mellitus type 1 or diabetes mellitus type 1).ab,kf,ti.
26	Arthritis, Juvenile/
27	(Arthritis or juvenile idiopathic arthritis or juvenile arthritis or arthritis, juvenile or idiopathic arthritis, juvenile or arthritis, juvenile idiopathic or juvenile chronic arthritis or chronic arthritis, juvenile or arthritis, juvenile chronic or juvenile rheumatoid arthritis or arthritis, juvenile rheumatoid or rheumatoid arthritis, juvenile or childhood arthritis or arthritis, childhood or arthritides, childhood or childhood arthritides or systemic arthritis, juvenile or arthritis, juvenile systemic or juvenile systemic arthritis or Rheumatoid Factor Positive Juvenile Polyarthritis or Rheumatoid Factor Negative Juvenile Polyarthritis or polyarthritis, juvenile, rheumatoid factor negative or polyarthritis, juvenile, rheumatoid factor positive or polyarticular juvenile idiopathic arthritis or pcjia or pjia polyarticular juvenile idiopathic arthritis or polyarticular-course juvenile idiopathic arthritis or JIA or juvenile arthritis deformans or arthritis deformans juvenilis or juvenile arthropathy or arthropathy, juvenile or reactive arthritis or Arthritis reactive).ab,kf,ti.
28	22 or 23 or 24 or 25 or 26 or 27
29	Social Networking/ or Social Support/
30	(Community health service* or health service*, community or service*, community health or community health care or health care, community or community healthcare or healthcare, community or Social Support or social care or Communit* or neighborhood* or Community Support* or Support Community or Community Network* or Community Care Network* or Network*, Community Care or Care Network*, Community or network*, community care or Community Health Network* or Health Network*, Community or Network*, Community Health or Community Participation or Participation, Community or Involvement*, Community or Community Involvement* or Community Action* or Action*, Community or Social Prescribing or Prescribing, Social or School health or school health service* or health service*, school or service*, school health or school-based health service* or health service*, school-based or service*, school-based health or school based health service* or health service*, school based or service*, school based health or school health promotion* or health promotion*, school or promotion*, school health or community-based participatory research or community based participatory research or participatory research, community-based or participatory research, community based or community care or community care service or community health action or community health care or participatory research or social network* or network*, social or online social network* or social network*, online or intervention*, school based or intervention*, school-based).ab,kf,ti.
31	13 or 14 or 15 or 16 or 17 or 18 or 19 or 20 or 21 or 29 or 30
32	Parents/
33	Siblings/
34	(((Family or families or family health or family research or family nursing* or nursing*, family or family-centered nursing* or family centered nursing* or nursing*, family-centered or nursing*, family centered or pediatric nursing* or nursing*, pediatric or children) and young people's nursing) or school nursing* or nursing*, school or research family or family support* or support family or dyad* or family-based or family-centered or family-oriented or family-anchored or family functioning or family coping or family based or family centered or family oriented or family

	anchored or family centered care or family interaction or family intervention* or family member* or Parent* or Sibling*).ab,kf,ti.
35	1 or 2 or 3 or 4 or 5 or 32 or 33 or 34
36	12 and 35
37	31 and 36
38	28 and 37

EMBASE

Embase Classic+Embase <1947 to 2025 September 15>	
1	child health care/ or child/ or child care/ or child health/
2	adolescent health/ or adolescent/
3	(Child* or child health or Child Health Care or child health services or Rehabilitation pediatric or pediatric rehabilitation or Adolescen* or Adolescent Health or adolescent health services or youth* or teen*).ab,kf,ti.
4	1 or 2 or 3
5	family nursing/ or family support/ or family centered care/ or family/ or family health/
6	family health/
7	family/ or family centered care/
8	family support/ or family/
9	family/ or family nursing/
10	community care/
11	community support/ or community care/ or social support/
12	social support/
13	community health nursing/
14	community care/ or community participation/
15	social prescribing/
16	school health service/
17	participatory research/
18	asthma/
19	(Asthma* or bronchial asthma or asthma bronchial* or asthma pulmonal* or chronic asthma or lung allerg* or asthma, bronchial or asthmatic or bronchus asthma or childhood asthma or early onset asthma).ab,kf,ti.
20	diabetes mellitus/
21	(diabetes mellitus, type 1 or type 1 diabetes or insulin-dependent diabetes mellitus or juvenile-onset diabetes mellitus or insulin-dependent diabetes mellitus 1 or juvenile-onset diabetes or diabetes type 1 or childhood diabetes or diabetes, type 1 or diabetes mellitus, insulin-dependent or diabetes mellitus, insulin dependent diabetes mellitus, juvenile-onset or diabetes mellitus, juvenile onset or diabetes mellitus, type I or diabetes mellitus, insulin-dependent, 1 or insulin dependent diabetes mellitus 1 or diabetes, juvenile-onset or juvenile onset diabetes or diabetes, autoimmune or autoimmune diabetes or diabetes mellitus, brittle or type 1 diabetes mellitus or Diabetes, Autoimmune or Autoimmune Diabetes or iddm or Diabetes Mellitus, Insulin-Dependent or Diabetes Mellitus, Insulin-Dependent, 1 or Diabetes, Type 1 or Insulin Dependent Diabetes Mellitus 1 or insulin dependent diabetes mellitus or juvenile onset diabetes mellitus or early onset diabetes mellitus or diabetes mellitus type 1 or diabetes mellitus type 1).ab,kf,ti.

22	juvenile rheumatoid arthritis/
23	(Arthritis or juvenile idiopathic arthritis or juvenile arthritis or arthritis, juvenile or idiopathic arthritis, juvenile or arthritis, juvenile idiopathic or juvenile chronic arthritis or chronic arthritis, juvenile or arthritis, juvenile chronic or juvenile rheumatoid arthritis or arthritis, juvenile rheumatoid or rheumatoid arthritis, juvenile or childhood arthritis or arthritis, childhood or arthritides, childhood or childhood arthritides or systemic arthritis, juvenile or arthritis, juvenile systemic or juvenile systemic arthritis or Rheumatoid Factor Positive Juvenile Polyarthritis or Rheumatoid Factor Negative Juvenile Polyarthritis or polyarthritis, juvenile, rheumatoid factor negative or polyarthritis, juvenile, rheumatoid factor positive or polyarticular juvenile idiopathic arthritis or pcjia or pjia polyarticular juvenile idiopathic arthritis or polyarticular-course juvenile idiopathic arthritis or JIA or juvenile arthritis deformans or arthritis deformans juvenilis or juvenile arthropathy or arthropathy, juvenile or reactive arthritis or Arthritis reactive).ab,kf,ti.
24	18 or 19 or 20 or 21 or 22 or 23
25	social network/
26	online social network/ or social network/ or social support/
27	(Community health service* or health service*, community or service*, community health or community health care or health care, community or community healthcare or healthcare, community or Social Support or social care or Communit* or neighborhood* or Community Support* or Support Community or Community Network* or Community Care Network* or Network*, Community Care or Care Network*, Community or network*, community care or Community Health Network* or Health Network*, Community or Network*, Community Health or Community Participation or Participation, Community or Involvement*, Community or Community Involvement* or Community Action* or Action*, Community or Social Prescribing or Prescribing, Social or School health or school health service* or health service*, school or service*, school health or school-based health service* or health service*, school-based or service*, school-based health or school based health service* or health service*, school based or service*, school based health or school health promotion* or health promotion*, school or promotion*, school health or community-based participatory research or community based participatory research or participatory research, community-based or participatory research, community based or community care or community care service or community health action or community health care or participatory research or social network* or network*, social or online social network* or social network*, online or intervention*, school based or intervention*, school-based).ab,kf,ti.
28	10 or 11 or 12 or 13 or 14 or 15 or 16 or 17 or 25 or 26 or 27
29	parent/
30	sibling/
31	(((Family or families or family health or family research or family nursing* or nursing*, family or family-centered nursing* or family centered nursing* or nursing*, family-centered or nursing*, family centered or pediatric nursing* or nursing*, pediatric or children) and young people's nursing) or school nursing* or nursing*, school or research family or family support* or support family or dyad* or family-based or family-centered or family-oriented or family-anchored or family functioning or family coping or family based or family centered or family oriented or family anchored or family centered care or family interaction or family intervention* or family member* or Parent* or Sibling*).ab,kw,ti.
32	5 or 6 or 7 or 8 or 9 or 29 or 30 or 31
33	4 and 32
34	28 and 33
35	24 and 34

PsychINFO

APA PsycInfo <1806 to September 2025 Week 2>	
1	exp Community Health/
2	exp Community Health/ or exp Communities/
3	exp Social Support/ or exp Community Health/ or exp Communities/ or exp Community Involvement/
4	exp Community Health/ or exp Communities/
5	exp Online Social Networks/ or exp Social Networks/ or exp Communities/ or exp Community Involvement/ or exp Social Support/
6	exp Community Involvement/ or exp Community Health/
7	exp Social Prescribing/
8	exp School Based Intervention/
9	exp Community-Based Participatory Research/
10	exp Community Involvement/
11	exp Online Social Networks/
12	exp School Based Intervention/
13	(Community health service* or health service*, community or service*, community health or community health care or health care, community or community healthcare or healthcare, community or Social Support or social care or Communit* or neighborhood* or Community Support* or Support Community or Community Network* or Community Care Network* or Network*, Community Care or Care Network*, Community or network*, community care or Community Health Network* or Health Network*, Community or Network*, Community Health or Community Participation or Participation, Community or Involvement*, Community or Community Involvement* or Community Action* or Action*, Community or Social Prescribing or Prescribing, Social or School health or school health service* or health service*, school or service*, school health or school-based health service* or health service*, school-based or service*, school-based health or school based health service* or health service*, school based or service*, school based health or school health promotion* or health promotion*, school or promotion*, school health or community-based participatory research or community based participatory research or participatory research, community-based or participatory research, community based or community care or community care service or community health action or community health care or participatory research).ab,ti,tw.
14	1 or 2 or 3 or 4 or 5 or 6 or 7 or 8 or 9 or 10 or 11 or 12 or 13
15	exp Child Care/ or exp Child Support/ or exp Child Health/
16	exp Child Care/ or exp Child Health/
17	exp Child Support/
18	exp Adolescent Health/
19	(Child* or child health or Child Health Care or child health services or Rehabilitation pediatric or pediatric rehabilitation or Adolescen* or Adolescent Health or adolescent health services or youth* or teen*).ab,ti,tw.
20	1 or 2 or 3 or 4 or 5 or 6 or 7 or 8 or 9 or 10 or 11 or 12 or 13 or 15 or 16 or 17 or 18 or 19
21	exp Family/ or exp Family Intervention/
22	exp Family/ or exp Family Members/
23	exp Family Intervention/
24	exp Family Members/
25	exp Asthma/

26	(Asthma* or bronchial asthma or asthma bronchial* or asthma pulmonal* or chronic asthma or lung allerg* or asthma, bronchial or asthmatic or bronchus asthma or childhood asthma or early onset asthma).ab,ti,tw.
27	exp Type 1 Diabetes/
28	(diabetes mellitus, type 1 or type 1 diabetes or insulin-dependent diabetes mellitus or juvenile-onset diabetes mellitus or insulin-dependent diabetes mellitus 1 or juvenile-onset diabetes or diabetes type 1 or childhood diabetes or diabetes, type 1 or diabetes mellitus, insulin-dependent or diabetes mellitus, insulin dependent diabetes mellitus, juvenile-onset or diabetes mellitus, juvenile onset or diabetes mellitus, type I or diabetes mellitus, insulin-dependent, 1 or insulin dependent diabetes mellitus 1 or diabetes, juvenile-onset or juvenile onset diabetes or diabetes, autoimmune or autoimmune diabetes or diabetes mellitus, brittle or type 1 diabetes mellitus or Diabetes, Autoimmune or Autoimmune Diabetes or iddm or Diabetes Mellitus, Insulin-Dependent or Diabetes Mellitus, Insulin-Dependent, 1 or Diabetes, Type 1 or Insulin Dependent Diabetes Mellitus 1 or insulin dependent diabetes mellitus or juvenile onset diabetes mellitus or early onset diabetes mellitus or diabetes mellitus type 1 or diabetes mellitus type 1).ab,ti,tw.
29	(Arthritis or juvenile idiopathic arthritis or juvenile arthritis or arthritis, juvenile or idiopathic arthritis, juvenile or arthritis, juvenile idiopathic or juvenile chronic arthritis or chronic arthritis, juvenile or arthritis, juvenile chronic or juvenile rheumatoid arthritis or arthritis, juvenile rheumatoid or rheumatoid arthritis, juvenile or childhood arthritis or arthritis, childhood or arthritides, childhood or childhood arthritides or systemic arthritis, juvenile or arthritis, juvenile systemic or juvenile systemic arthritis or Rheumatoid Factor Positive Juvenile Polyarthritis or Rheumatoid Factor Negative Juvenile Polyarthritis or polyarthritis, juvenile, rheumatoid factor negative or polyarthritis, juvenile, rheumatoid factor positive or polyarticular juvenile idiopathic arthritis or pcjia or pjia polyarticular juvenile idiopathic arthritis or polyarticular-course juvenile idiopathic arthritis or JIA or juvenile arthritis deformans or arthritis deformans juvenilis or juvenile arthropathy or arthropathy, juvenile or reactive arthritis or Arthritis reactive).ab,ti,tw.
30	25 or 26 or 27 or 28 or 29
31	exp Parents/
32	exp Siblings/
33	(((Family or families or family health or family research or family nursing* or nursing*, family or family-centered nursing* or family centered nursing* or nursing*, family-centered or nursing*, family centered or pediatric nursing* or nursing*, pediatric or children) and young people's nursing) or school nursing* or nursing*, school or research family or family support* or support family or dyad* or family-based or family-centered or family-oriented or family-anchored or family functioning or family coping or family based or family centered or family oriented or family anchored or family centered care or family interaction or family intervention* or family member* or Parent* or Sibling*).ab,ti,tw.
34	21 or 22 or 23 or 24 or 31 or 32 or 33
35	20 and 34
36	14 and 35
37	30 and 36

Cochrane

Date Run:	16-09-2025 20:01
ID	Search
#1	MeSH descriptor: [Child] explode all trees
#2	MeSH descriptor: [Adolescent] explode all trees

#3	MeSH descriptor: [Child Health] explode all trees
#4	MeSH descriptor: [Child Health Services] explode all trees
#5	MeSH descriptor: [Child Care] explode all trees
#6	MeSH descriptor: [Adolescent Health] explode all trees
#7	MeSH descriptor: [Adolescent Health Services] explode all trees
#8	(Child* OR child health OR Child Health Care OR child health services OR Rehabilitation pediatric OR pediatric rehabilitation OR Adolescen* OR Adolescent Health OR adolescent health services OR youth* OR teen*):ti
#9	(Child* OR child health OR Child Health Care OR child health services OR Rehabilitation pediatric OR pediatric rehabilitation OR Adolescen* OR Adolescent Health OR adolescent health services OR youth* OR teen*):ab
#10	(Child* OR child health OR Child Health Care OR child health services OR Rehabilitation pediatric OR pediatric rehabilitation OR Adolescen* OR Adolescent Health OR adolescent health services OR youth* OR teen*):kw
#11	#1 or #2 or #3 or #4 or #5 or #5 or #6 or #7 or #8 or #9 or #10
#12	MeSH descriptor: [Family] explode all trees
#13	MeSH descriptor: [Family Health] explode all trees
#14	MeSH descriptor: [Family Support] explode all trees
#15	MeSH descriptor: [Family Nursing] explode all trees
#16	MeSH descriptor: [Parents] explode all trees
#17	MeSH descriptor: [Siblings] explode all trees
#18	(Family OR families OR family health OR family research OR family nursing* OR nursing*, family OR family-centered nursing* OR family centered nursing* OR nursing*, family-centered OR nursing*, family centered OR pediatric nursing* OR nursing*, pediatric OR children and young people's nursing OR school nursing* OR nursing*, school OR research family OR family support* OR support family OR dyad* OR family-based OR family-centered OR family-oriented OR family-anchored OR family functioning OR family coping OR family based OR family centered OR family oriented OR family anchored OR family centered care OR family interaction OR family intervention* OR family member* OR Parent* OR Sibling*):ti,ab,kw
#19	#12 or #13 or #14 or #15 or #16 or #17 or #18
#20	MeSH descriptor: [Community Health Services] explode all trees
#21	MeSH descriptor: [Social Support] explode all trees
#22	MeSH descriptor: [Community Support] explode all trees
#23	MeSH descriptor: [Social Networking] explode all trees
#24	MeSH descriptor: [Community Participation] explode all trees
#25	MeSH descriptor: [Social Prescribing] explode all trees
#26	MeSH descriptor: [School Health Services] explode all trees
#27	MeSH descriptor: [Community-Based Participatory Research] explode all trees
#28	(Community health service* OR health service*, community OR service*, community health OR community health care OR health care, community OR community healthcare OR healthcare, community OR Social Support OR social care OR Communit* OR

	neighborhood* OR Community Support* OR Support Community OR Community Network* OR Community Care Network* OR Network*, Community Care OR Care Network*, Community OR network*, community care OR Community Health Network* OR Health Network*, Community OR Network*, Community Health OR Community Participation OR Participation, Community OR Involvement*, Community OR Community Involvement* OR Community Action* OR Action*, Community OR Social Prescribing OR Prescribing, Social OR School health OR school health service* OR health service*, school OR service*, school health OR school-based health service* OR health service*, school-based OR service*, school-based health OR school based health service* OR health service*, school based OR service*, school based health OR school health promotion* OR health promotion*, school OR promotion*, school health OR community-based participatory research OR community based participatory research OR participatory research, community-based OR participatory research, community based OR community care OR community care service OR community health action OR community health care OR participatory research OR social network* OR network*, social OR online social network* OR social network*, online OR intervention*, school based OR intervention*, school-based):ti,ab,kw
#29	#20 or #21 or #22 or #23 or #24 or #25 or #26 or #27 or #28
#30	MeSH descriptor: [Asthma] explode all trees
#31	(Asthma* OR bronchial asthma OR asthma bronchial* OR asthma pulmonal* OR chronic asthma OR lung allerg* OR asthma, bronchial OR asthmatic OR bronchus asthma OR childhood asthma OR early onset asthma):ti
#32	(Asthma* OR bronchial asthma OR asthma bronchial* OR asthma pulmonal* OR chronic asthma OR lung allerg* OR asthma, bronchial OR asthmatic OR bronchus asthma OR childhood asthma OR early onset asthma):ab
#33	(Asthma* OR bronchial asthma OR asthma bronchial* OR asthma pulmonal* OR chronic asthma OR lung allerg* OR asthma, bronchial OR asthmatic OR bronchus asthma OR childhood asthma OR early onset asthma):kw
#34	MeSH descriptor: [Diabetes Mellitus, Type 1] explode all trees
#35	(diabetes mellitus, type 1 OR type 1 diabetes OR insulin-dependent diabetes mellitus OR juvenile-onset diabetes mellitus OR insulin-dependent diabetes mellitus 1 OR juvenile-onset diabetes OR diabetes type 1 OR childhood diabetes OR diabetes, type 1 OR diabetes mellitus, insulin-dependent OR diabetes mellitus, insulin dependent diabetes mellitus, juvenile-onset OR diabetes mellitus, juvenile onset OR diabetes mellitus, type I OR diabetes mellitus, insulin-dependent, 1 OR insulin dependent diabetes mellitus 1 OR diabetes, juvenile-onset OR juvenile onset diabetes OR diabetes, autoimmune OR autoimmune diabetes OR diabetes mellitus, brittle OR type 1 diabetes mellitus OR Diabetes, Autoimmune OR Autoimmune Diabetes OR iddm OR Diabetes Mellitus, Insulin-Dependent OR Diabetes Mellitus, Insulin-Dependent, 1 OR Diabetes, Type 1 OR Insulin Dependent Diabetes Mellitus 1 OR insulin dependent diabetes mellitus OR juvenile onset diabetes mellitus OR early onset diabetes mellitus OR diabetes mellitus type 1 OR diabetes mellitus type 1):ti,ab,kw
#36	MeSH descriptor: [Arthritis, Juvenile] explode all trees
#37	(Arthritis OR juvenile idiopathic arthritis OR juvenile arthritis OR arthritis, juvenile OR idiopathic arthritis, juvenile OR arthritis, juvenile idiopathic OR juvenile chronic arthritis OR chronic arthritis, juvenile OR arthritis, juvenile chronic OR juvenile rheumatoid arthritis OR arthritis, juvenile rheumatoid OR rheumatoid arthritis, juvenile OR childhood arthritis OR arthritis, childhood OR arthritides, childhood OR childhood arthritides OR systemic arthritis, juvenile OR arthritis, juvenile systemic OR juvenile systemic arthritis OR Rheumatoid Factor Positive Juvenile Polyarthritis OR Rheumatoid Factor Negative Juvenile Polyarthritis OR polyarthritis, juvenile, rheumatoid factor negative OR polyarthritis, juvenile, rheumatoid factor positive OR polyarticular juvenile idiopathic arthritis OR pcjia OR pjia polyarticular

	juvenile idiopathic arthritis OR polyarticular-course juvenile idiopathic arthritis OR JIA OR juvenile arthritis deformans OR arthritis deformans juvenilis OR juvenile arthropathy OR arthropathy, juvenile OR reactive arthritis OR Arthritis reactive):ti,ab,kw
#38	#30 or #31 or #34 or #35 or #36 or #37
#39	#11 and #19
#40	#39 and #29
#41	#40 and #38 in Cochrane Reviews, Cochrane Protocols, Trials

CINAHL

Date: 16.09.2025

((((MH "Child" OR MH "Child Health" OR MH "Child Care" OR MH "Child Health Services") OR (MH "Adolescence" OR MH "Adolescent Health" OR MH "Adolescent Health Services") OR MH "Rehabilitation, Pediatric" OR XB (Child* OR child health OR Child Health Care OR child health services OR Rehabilitation pediatric OR pediatric rehabilitation OR Adolescen* OR Adolescent Health OR adolescent health services OR youth* OR teen*)) AND ((MH "Family" OR MH "Family Support" OR MH "Family Health") OR (MH "Family Centered Care" OR MH "Family") OR (MH "Family Nursing" OR MH "Family Nurses") OR MH "Family Support" OR MH "Parents" OR MH "Siblings" OR XB (Family OR families OR family health OR family research OR family nursing* OR nursing*, family OR family-centered nursing* OR family centered nursing* OR nursing*, family-centered OR nursing*, family centered OR pediatric nursing* OR nursing*, pediatric OR children and young people's nursing OR school nursing* OR nursing*, school OR research family OR family support* OR support family OR dyad* OR family-based OR family-centered OR family-oriented OR family-anchored OR family functioning OR family coping OR family based OR family centered OR family oriented OR family anchored OR family centered care OR family interaction OR family intervention* OR family member* OR Parent* OR Sibling*))) AND (MH "Community Health Services" OR (MH "Community Support" OR MH "Community Networks" OR MH "Community Health Workers") OR MH "Community Health Nursing" OR MH "Support, Social" OR MH "Social Networks" OR (MH "Social Networking" OR MH "Online Social Networking") OR MH "Social Participation" OR MH "School Health Services" OR MH "School Health" OR XB (Community health service* OR health service*, community OR service*, community health OR community health care OR health care, community OR community healthcare OR healthcare, community OR Social Support OR social care OR Communit* OR neighborhood* OR Community Support* OR Support Community OR Community Network* OR Community Care Network* OR Network*, Community Care OR Care Network*, Community OR network*, community care OR Community Health Network* OR Health Network*, Community OR Network*, Community Health OR Community Participation OR Participation, Community OR Involvement*, Community OR Community Involvement* OR Community Action* OR Action*, Community OR Social Prescribing OR Prescribing, Social OR School health OR school health service* OR health service*, school OR service*, school health OR school-based health service* OR health service*, school-based OR service*, school-based health OR school based health service* OR health service*, school based OR service*, school based health OR school health promotion* OR health promotion*, school OR promotion*, school health OR community-based participatory research OR community based participatory research OR participatory research, community-based OR participatory research, community based OR community care OR community care service OR community health action OR community health care OR participatory research OR social network* OR network*, social OR online social network* OR social

network*, online OR intervention*, school based OR intervention*, school-based))) AND (MH "Asthma" OR MH "Diabetes Mellitus, Type 1" OR (MH "Reactive Arthritis" OR MH "Arthritis, Juvenile Rheumatoid") OR XB (Asthma* OR bronchial asthma OR asthma bronchial* OR asthma pulmonal* OR chronic asthma OR lung allerg* OR asthma, bronchial OR asthmatic OR bronchus asthma OR childhood asthma OR early onset asthma) OR TX (Asthma* OR bronchial asthma OR asthma bronchial* OR asthma pulmonal* OR chronic asthma OR lung allerg* OR asthma, bronchial OR asthmatic OR bronchus asthma OR childhood asthma OR early onset asthma) OR XB (diabetes mellitus, type 1 OR type 1 diabetes OR insulin-dependent diabetes mellitus OR juvenile-onset diabetes mellitus OR insulin-dependent diabetes mellitus 1 OR juvenile-onset diabetes OR diabetes type 1 OR childhood diabetes OR diabetes, type 1 OR diabetes mellitus, insulin-dependent OR diabetes mellitus, insulin dependent diabetes mellitus, juvenile-onset OR diabetes mellitus, juvenile onset OR diabetes mellitus, type I OR diabetes mellitus, insulin-dependent, 1 OR insulin dependent diabetes mellitus 1 OR diabetes, juvenile-onset OR juvenile onset diabetes OR diabetes, autoimmune OR autoimmune diabetes OR diabetes mellitus, brittle OR type 1 diabetes mellitus OR Diabetes, Autoimmune OR Autoimmune Diabetes OR iddm OR Diabetes Mellitus, Insulin-Dependent OR Diabetes Mellitus, Insulin-Dependent, 1 OR Diabetes, Type 1 OR Insulin Dependent Diabetes Mellitus 1 OR insulin dependent diabetes mellitus OR juvenile onset diabetes mellitus OR early onset diabetes mellitus OR diabetes mellitus type 1 OR diabetes mellitus type 1))

Google Scholar

Date: 16.09.2025

((Child* OR adolescent*) and (Family OR families or Parent* or Sibling*) and (Communit* OR Network* or "Social support" OR "Social prescribing" Or school*) and (Asthma OR "Type 1 Diabetes" OR "juvenile arthritis"))

Search link: https://scholar.google.com/scholar?scilib=1032&hl=da&as_sdt=0,5&scioq=((Child*+OR+adolescent*)+and+(Family+OR+families+or+Parent*+or+Sibling*)+and+(Communit*+OR+Network*+or+%22Social+support%22+OR+%22Social+prescribing%22+Or+school*)+and+(Asthma+OR+%22Type+1+Diabetes%22+OR+%22juvenile+arthritis%22))

Sigle-OGrey

Date: 16.09.2025

((Child* OR adolescent*) and (Family OR families or Parent* or Sibling*) and (Communit* OR Network* or "Social support" OR "Social prescribing" Or school*) and (Asthma OR "Type 1 Diabetes" OR "juvenile arthritis"))
